# Methylsulfonylmethane Attenuates Dexamethasone-Induced Hepatic Insulin Resistance in Rats: Associations with SGK1, p-AMPK/mTOR, Inflammatory and Angiogenic Markers

**DOI:** 10.3390/jox16040121

**Published:** 2026-06-30

**Authors:** Ahmad A. Alresheedi, Omnia A. Nour, Dalia H. El-Kashef, Manar A. Nader

**Affiliations:** 1 Department of Pharmacology and Toxicology, Faculty of Pharmacy, Mansoura University, Mansoura 35516, Egypt; aalresheedi@gmail.com (A.A.A.); omnianour89@mans.edu.eg (O.A.N.); dalia_elkashef@mans.edu.eg (D.H.E.-K.); 2 Faculty of Health Science Technology, Mansoura National University, Gamasa 7731168, Egypt

**Keywords:** methylsulfonylmethane, dexamethasone, insulin resistance, SGK1 signaling, NLRP3 inflammasome, AMPK/mTOR pathway

## Abstract

**Background/Objectives:** Glucocorticoid therapy remains clinically indispensable, yet its long-term use is profoundly constrained by insulin resistance (IR), hepatic steatosis, and progressive metabolic dysfunction. Methylsulfonylmethane (MSM), a naturally occurring sulfur-containing nutraceutical with established antioxidant and anti-inflammatory activities, has emerged as a promising metabolic modulator; however, its therapeutic relevance in glucocorticoid-induced hepatic IR has not previously been explored. **Methods:** Male Wistar rats received MSM (200 or 400 mg/kg/day, p.o.) for 14 days, while dexamethasone (DEX) (8 mg/kg/day, i.p.) was administered during the final 7 days to induce severe metabolic dysfunction. **Results:** DEX provoked profound IR, dyslipidemia, oxidative stress, hepatocellular injury, and steatotic degeneration accompanied by marked ultrastructural abnormalities. Remarkably, MSM conferred dose-dependent metabolic and hepatoprotective effects, significantly restoring glucose homeostasis, insulin responsiveness, lipid metabolism, and hepatic structural integrity. Mechanistically, MSM exerted a pleiotropic regulatory effect through suppression of the glucocorticoid-responsive kinase SGK1, restoration of AMPK/mTOR signaling balance, and normalization of insulin signaling pathways and metabolic transcriptional regulators. Furthermore, MSM effectively attenuated oxidative stress and inflammatory amplification consistent with modulation of the NLRP3/NF-κB/IL-6 axis. Importantly, the current work identifies angiogenic remodeling demonstrated by DEX-induced upregulation of VEGF and CD34, both of which were substantially suppressed by MSM treatment. **Conclusions:** This study provides novel evidence that MSM mitigates glucocorticoid-induced hepatic IR through coordinated modulation of glucocorticoid-responsive kinases, metabolic signaling networks, redox–inflammatory cascades, and pathological angiogenesis. Consequently, MSM may represent a promising candidate for further preclinical and clinical evaluation regarding its capacity to limit glucocorticoid-associated metabolic burdens.

## 1. Introduction

Insulin resistance (IR) is a pathological disorder characterized by a reduced response to systemic insulin by various tissues and organs, including skeletal muscles, fat tissues, and the liver, leading to hyperglycemia and metabolic syndrome [1,2]. Although the hyperglycemic response leads to enhanced secretion of insulin by β-cells, the insulin-insensitive cells persistently show inadequate response, culminating in subversion of glucose homeostasis [3]. This homeostatic disruption triggers the compensatory mechanism involving enhanced production of glucose by the liver through augmentation of glycogenolysis and gluconeogenesis [4,5]. This further complicates glucose metabolism by elevating plasma glucose levels, resulting in a chronic hyperglycemic state [6].

Dexamethasone (DEX), a synthetic glucocorticoid analogue, is extensively used for the long-term management of inflammatory and immune disorders. However, its therapeutic utility is often limited by debilitating metabolic adverse effects characterized by systemic insulin resistance and hyperglycemia due to excessive reactive oxygen species (ROS) and oxidative stress, leading to acute tissue injuries [7]. Administration of DEX to animals induces acute liver injury (ALI) and develops a well-recognized in vivo model for the investigation of fatty liver degeneration and hepatic steatosis. Being a glucocorticoid, DEX enhances the buildup of fat into the hepatocytes by augmenting the synthesis of fatty acids while hampering β-oxidation of them simultaneously [8]. Apart from metabolic derangements, DEX also exerts hepatic tissue injury through various cellular mechanisms such as autophagy-related disorders and pyroptosis, leading to extensive damage to liver architecture [9]. Furthermore, one of the significant complications associated with DEX administration is the formation of liver steatosis progressing towards the development of early non-alcoholic fatty liver diseases (NAFLDs) [10]. If left untreated, this early phase of NAFLD may progress to liver fibrosis and irreversible liver injury. IR has been determined as a key factor in critical mechanisms involving glucocorticoid-related liver steatosis. Additionally, DEX application may also induce steroid-related diabetes by exerting metabolic dysfunctions caused by β-cell malformations and IR at tissue levels [11,12].

Methylsulfonylmethane (MSM) is an organosulfur agent occurring naturally in various plants and has been extensively used for its antioxidant and anti-inflammatory properties as a food supplement and part of complementary and alternative medicine (CAM) [13]. Many current research findings highlight that MSM produces beneficial pharmacological actions in the management of hyperlipidemia, insulin resistance, hepatic steatosis, and other metabolic derangements [14]. The core mechanisms for these actions include the diminution of oxidative stress markers, pro-inflammatory cytokines (e.g., TNF-α, NF-κβ, and IL-6), and modulation of major metabolic signaling mechanisms [15]. The therapeutic significance of MSM has also been documented in metabolism-associated fatty liver disease (MAFLD) through restoration of autophagy consistent with modulation of the AMPK/mTOR/ULK1 axis [16].

To the best of our knowledge, the protective actions and associated mechanisms of MSM against DEX-induced insulin resistance and metabolic dysfunctions remain unexplored. Hence, we hypothesized that MSM may interrupt DEX-induced systemic insulin resistance and accompany hyperglycemic defects in the murine model.

## 2. Materials and Methods

### 2.1. Drugs and Chemicals

DEX in the form of dexamethasone sodium phosphate has been acquired from the market as a commonly available formulation manufactured by Amriya Pharmaceutical Industries (Alexandria, Egypt) as ampules of 8 mg/2 mL, USP^®^, and diluted before the injection in sterile 0.9% normal saline. MSM was supplied by (Sigma-Aldrich, St. Louis, MO, USA, #PHR1346-1G) and mixed in 0.5% (*w*/*v*) carboxymethylcellulose (CMC) solution for the purpose of oral gavage. All other chemicals consumed are of the finest grade.

### 2.2. Animals

Adult Wistar male rats having weights of 200 ± 20 g were recruited for this study. The rats were supplied by the Vacsera (an Egyptian Organization for Bio-Products and Vaccines, Giza, Egypt). After receiving consignment, the rats were kept for acclimatization for seven days under a standardized laboratory environment: a temperature of 25 ± 2 °C was used, with 12 h light/dark phases, and a relative humidity kept around 50–60%. Animals were segregated randomly into groups of six rats per cage with unlimited access to water and rodent chow. All procedures, including laboratory animal welfare, were approved by the Mansoura University Animal Care and Use Committee (MU-ACUC), recording Protocol No: RHARM.PhD.24.06.43. Also, all experiments were designed and reported in accordance with the Design and Analysis guidelines described by Curtis et al. (BJP).

### 2.3. Study Design

Animals were randomly allocated to experimental groups using a computer-generated randomization schedule. Investigators performing outcome assessments and data analysis were blinded to group allocation. A total of thirty male Wistar rats were randomly grouped into five groups as follows: Control group: Rats received no treatment except vehicle for 14 days consecutively. MSM400 group: Rats were administered MSM dose alone (400 mg/kg/day, orally) [17] for 14 days consecutively [16]. DEX group: Rats administered CMC for 7 days as a drug vehicle, then injected DEX (i.p.) at 8 mg/kg/day dosage for 7 days consecutively, starting on day 8 of the trial [18]. MSM200 + DEX group: Rats were first treated with an oral dose of MSM (200 mg/kg/day) [17,19] for 14 days consecutively. On day 8 of trial, DEX (8 mg/kg/day, i.p.) was administered 60 min after MSM administration and continued for the last 7 days consecutively. MSM400 + DEX group: Rats were first treated with an oral dose of MSM (400 mg/kg/day) [17] for 14 days consecutively. On day 8 of trial, DEX (8 mg/kg/day, i.p.) was administered 60 min after MSM administration and continued for the last 7 days consecutively.

Body weights of rats were measured regularly, along with monitoring of distressing signs throughout the trial period. For all days of the experimental period, the body weights of all rats were noted using a calibrated weighing balance. The change in body weight of each animal was documented as a percentage variation in the initial weight by calculating the difference between the primary body weights (recorded on day 1) and the final body weights (recorded on the last day). The blood samples were extracted from the venous plexus of the retro-orbital region employing a slight anesthetic agent secobarbital (50 mg/kg, i.p.). The blood samples were allowed to clot before centrifugation for 15 min at a rate of 3000 rpm, maintaining 4 °C to extract serum, which was stored at −20 °C for the purpose of biochemical investigation. Following the collection of blood samples, the animals were ethically euthanized by performing cervical dislocation. Livers of all rats were excised, rinsed with ice-cold saline to remove blood material, blotted to dry, and weighed. Each liver was cut into three pieces: one part for histopathological and immunohistochemical (IHC) investigation was fixed in 10% solution of neutral-buffered formalin, the second part was kept in a suitable buffer for transmission electron microscopy (TEM), the third part was used for biochemical experimentation and flow cytometry analysis and the tissue homogenate was stored at −80 °C.

### 2.4. Oral Glucose Tolerance Test (OGTT)

To investigate the glucose tolerance of the rats, the OGTT was implemented on the last day of experimentation. Following a 10 h overnight of fasting, during which they were provided unrestricted access to water, each rat received an oral solution of 40% glucose mixture (2 g/kg bw) [20]. The levels of blood glucose were monitored using a glucometer at 0 min fast and 30, 60, 120, and 180 min after oral intake of glucose solution. The tail-vein puncture was used to collect blood samples for the determination of blood glucose levels at baseline (0 min, prior to glucose administration), followed by 30, 60, 120, and 180 min post-glucose employing a recognized, validated glucometer device (Accu-Chek^®^, Hoffmann-La Roche diabetic care, Basel, Switzerland). The data was analyzed to evaluate the glucose utilization rate and glucose tolerance.

### 2.5. Fasting Insulin Level Assessment

On the last day, the rats underwent fasting for the ten previous hours overnight, although ad libitum access to water was given to them. On the same day, the fasting blood glucose was measured by performing the tail vein puncture using the handheld glucometer of Accu-Chek Active (Hoffmann-La Roche, Basel, Switzerland), and fasting serum insulin was assayed with the Roche Elecsys insulin assay kit (Roche Diagnostics, Mannheim, Germany, Cat. no. 12017547122).

### 2.6. Calculation of IR

The Homeostatic Model Assessment for IR (HOMA-IR) values were determined using the HOMA2 Calculator 2.2.2 software (Diabetes Trials Unit, University of Oxford, Oxford, UK) [21].

### 2.7. Determination of Liver Biomarkers

The liver function tests were employed to determine the pathological and metabolic status of the liver. The serum samples were analyzed for the detection of the liver biomarkers for aspartate alanine aminotransferase (ALT) and aminotransferase (AST) (AGAPPE Diagnostics Ltd., Kerala, India; Catalog # 11409005 and 11408005, respectively) to find out the intensity of hepatic impairment.

### 2.8. Assessment of Lipid Profile

Serum levels of triglycerides (TGs) and total cholesterol (TC) were evaluated enzymatically using commercially available chromogenic kits (Genesis, Obour City, Egypt; Cat. no. 1103102 and 1108101, respectively). High-density lipoprotein cholesterol (HDL-C) was quantified using an established precipitation-method kit (Biodiagnostic, Cairo, Egypt; Cat. No. HDL114100). Low-density lipoprotein cholesterol (LDL-C) was then estimated using the validated Friedewald formula, where VLDL-C = TG/5, and LDL-C = TC − (HDL-C + VLDL-C) [22].

### 2.9. Determination of Oxidative Stress Markers

The levels of malondialdehyde (MDA) were detected for oxidative stress, while glutathione (GSH) and total antioxidant capacity (TAC) were determined for anti-oxidative activities in liver tissues following the instructions provided by the manufacturer of commercial kits available locally (Biodiagnostic, Egypt, Cat. no. MD2529, GR2511, TA2513, respectively).

### 2.10. Determination of Liver Protein Content

Liver protein content was quantified using the Genei protein determination kit (Cat. No. 2624800021730, Genei Laboratories Pvt. Ltd., Bangalore, India), according to the Bradford method [23].

### 2.11. Enzyme-Linked Immunosorbent Assay (ELISA)

Liver tissue samples were homogenized in phosphate-buffered saline (10% *w*/*v*, pH 7.5) using a mini handheld homogenizer (Omni International, Inc., Kennesaw, GA, USA). After centrifugation of tissue homogenates at 5000 rpm/4 °C for 15 min, the supernatants were collected and used for ELISA assessment.

Observing the manufacturer’s guidelines, the following proteins were identified using commercially available ELISA kits (Table 1).

### 2.12. Histopathology Analysis

Liver (formalin-fixed and paraffin-embedded) tissues, uniformly sectioned (5 µm), dehydrated and heated in 10 mM buffered (pH 6.0) sodium citrate for retrieval of antigens, were stained in hematoxylin and eosin (H&E) for histopathological examination. The histopathological investigation was conducted using an optical microscope (Optoscope FZCO, Dubai, United Arab Emirates) equipped with a 4K Ultra HD digital camera (1/1.8″ CMOS sensor, Touptek Photonics, Hangzhou, China). 

The camera was mounted to the microscope via a ToupTek FMA050 fixed adapter (ToupTek Photonics, Hangzhou, China) by an independent observer blinded to the types of experimental groups. Established semiquantitative criteria were implemented to score two vital characteristics of macrovesicular steatosis and inflammatory activity. The steatosis score was assigned on a scale of five, where 0 denotes no steatosis, 1 specifies ≤25% involvement, 2 indicates 25–50%, 3 represents 50–75%, and 4 implies ≥75% involvement. Similarly, the severity of inflammation was categorized on a four-tier scale, where 0 represents normal tissue architecture preserved, while 3 indicates severe, widespread inflammation. The scoring system was adapted from a previously validated NAFLD/NASH scoring method [24].

### 2.13. Immunohistochemistry (IHC)

IHC analysis was carried out by deparaffinizing and rehydrating tissue sections while blocking endogenous peroxidase activity in 3% H_2_O_2_ solution. Antigens were then retrieved, and 5% BSA was applied to block non-specific binding sites. The sections were subsequently incubated with respective primary monoclonal antibodies at 4 °C for 8 h overnight, targeting serum regulated glucocorticoids kinase 1 (SGK1; ELK Biotechnology, cataloge no. EM1264), mammalian target of Rapamycin (mTOR; ELK Biotechnology, cataloge no. ES2865), and Sterol Regulatory Element-Binding Protein-1 (SREBP-1; ELK Biotechnology, cataloge no. ES8814), followed by incubation in HRP-conjugated secondary anti-rabbit antibodies for IHC analysis. Sectioned slides were directly examined beneath a camera-fitted light microscope for histological changes, including steatosis, inflammation, and cell ballooning, while 3, 3′-diaminobenzidine tetrachloride (DAB) was applied for the formation of a detectable stain, and visualized under a microscope. assessed using ImageJ software (Version 1.54, NIH, Bethesda, MD, USA). For each group, 5 random fields were taken at a magnification of ×400. The area of positive expression was measured and expressed as the Fraction Area (%), representing the percentage of the stained area relative to the total area of the microscopic field.

### 2.14. Transmission Electron Microscopy (TEM)

The tissue samples obtained from the liver and preserved in glutaraldehyde (2.5%) and paraformaldehyde (2.5%) using phosphate buffer during the last 24 h were fixed in 1% osmium tetroxide, properly dried, and embedded in resin. The tissue sections were then set on copper grids and stained using lead citrate and uranyl acetate. Afterwards, the stained sections were scanned under a TEM (JEOL 2100, Tokyo, Japan) at the Electron Microscopic Unit at Mansoura University.

### 2.15. Flowcytometry Analysis for CD34

CD34 surface marker expression was investigated by flow cytometry via the direct staining method. Cell suspensions were prepared from tissue using Tris-EDTA buffer and adjusted to a concentration of 1 × 10^6^ cells/mL in PBS containing 1% bovine serum albumin (PBS/BSA). Aliquots of 100 µL cell suspension were incubated with anti-CD34 antibody (7–10 µL) for 30 min at room temperature in the dark. After incubation, cells were washed with PBS/BSA and centrifuged at 1500–2000 rpm for 5 min, then the supernatant was discarded. The cell pellet was resuspended in 200 µL of PBS containing paraformaldehyde for fixation prior to analysis. Flow cytometric acquisition was performed using BD Accuri C6 Flow Cytometer (Becton Dickinson, Sunnyvale, CA, USA) equipped with a 488 nm argon laser. A total of 20,000 events were collected per sample and analyzed using instrument software (BD Accuri C6 Software 1.0.26; Becton Dickinson, Sunnyvale, CA, USA). The principle of flow cytometry is based on measuring light scattering and fluorescence emission from cells as they pass individually through a focused laser beam, allowing quantitative detection of labeled surface markers [25,26,27].

### 2.16. Statistical Analysis

A priori sample size was determined based on previous studies and power analysis (power = 0.8, α = 0.05) to detect biologically relevant differences between groups. Each experimental group included a minimum of five independent biological replicates (*n* ≥ 4). Data were analyzed by the Shapiro–Wilk normality test for normality. The parametric data from different groups were presented as mean ± SD. Statistical analysis was executed using the conveniently available GraphPad Prism statistical package version 8.0 (GraphPad Software Inc., San Diego, CA, USA). Quantitative differences among groups were analyzed by one-way analysis of variance (ANOVA) or repeated measures ANOVA followed by post hoc Tukey–Kramer test for multiple comparisons. For non-parametric data, the Kruskal–Wallis test was executed with post hoc comparison by Dunn’s test. A *p* < 0.05 was considered statistically significant.

## 3. Results

### 3.1. Impact of MSM (200 and 400 mg/kg) on Blood Glucose, Insulin, HOMA-IR Index, and OGTT

**Figure 1:**
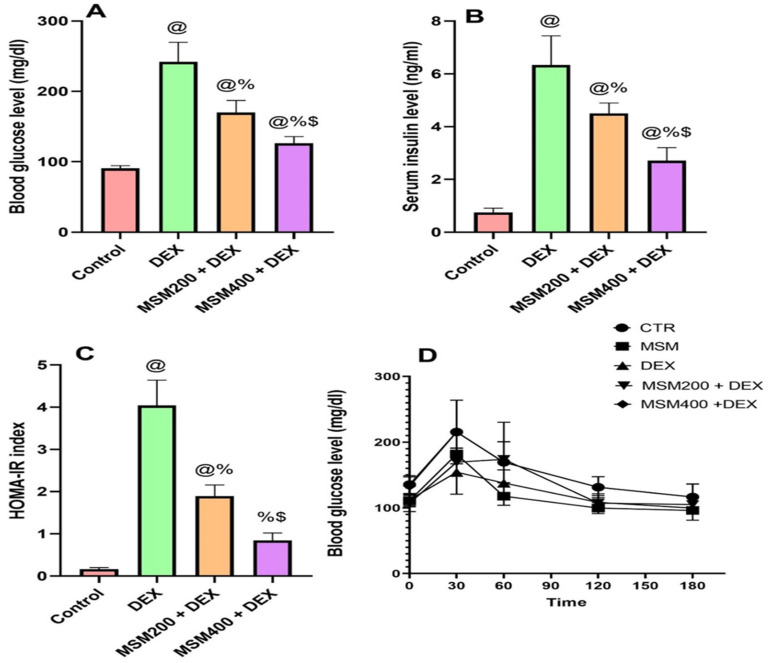
**Impact of MSM (200 and 400 mg/kg) on blood glucose, insulin, HOMA-IR index, and OGTT.** (**A**) Blood glucose level, (**B**) Serum insulin level, (**C**) HOMA-IR index, (**D**) Change in blood glucose level during the OGTT Data were expressed as mean ± SD, *n* = 4–6. @, %, $ *p* < 0.05 compared to the control, DEX, MSM200 + DEX, respectively, using one-way ANOVA for blood glucose, insulin, HOMA-IR index and repeated measures for OGTT, followed by the Tukey–Kramer multiple comparisons post hoc test. DEX: dexamethasone, HOMA-IR: Homeostatic Model Assessment of insulin resistance, MSM: methylsulfonylmethane, OGTT: oral glucose tolerance test.

Figure 1A showed that the blood glucose was significantly elevated in the rats administered with DEX (by 2.6-fold) compared to normal rats. It was noted that the addition of MSM at both doses (200 and 400 mg/kg) to DEX significantly reduced the serum glucose levels (by 29.6% and 47.8%, respectively). It was also observed that increased hyperglycemia induced by DEX led to increased levels of blood insulin (by 8.4-fold) (Figure 1B). On the other hand, when MSM was combined with DEX, the insulin blood levels were reduced (by 28.8% and 57.1%, respectively) in a dose-dependent manner at both MSM levels of 200 and 400 mg/kg. Moreover, the index of insulin resistance and beta-cell dysfunctions was represented by calculating HOMA-IR. It was found that HOMA-IR was markedly raised (by 24.3-fold) by DEX, demonstrating IR and beta-cell malfunctioning (Figure 1C). However, when MSM was added to the DEX application, it lowered the HOMA-IR index, accompanied by a reduction in glycemia (by 53.1% and 79%, respectively) in a dose-incremental way.

The OGTT was performed to determine the tolerance of excessive glucose intake. The results revealed that the rats given with 40% glucose solution and DEX illustrated impaired glucose tolerance as compared to the control group at specified time points (Figure 1D). Contrarily, combinatory treatment with MSM at 100 mg/kg significantly enhanced glucose consumption as characterized by lowered post-glucose levels. Furthermore, higher dosing at 400 mg/kg produced a profound reduction in blood glucose status compared to the DEX reference group, nearing the control group towards the last measurements of the test. These results exhibited the potential of MSM to reinstate the DEX-induced glucose values towards the normal status. Altogether, the control of fasting glucose, serum insulin, OGTT, and HOMA-IR index signified the beneficial effects of MSM in countering DEX-related disruptions of insulin activity and glucose utilization. Hence, MSM effectively reverses the hyperglycemic dysfunctions of DEX.

### 3.2. Impact of MSM (200 and 400 mg/kg) on Liver Function and Structure

**Figure 2:**
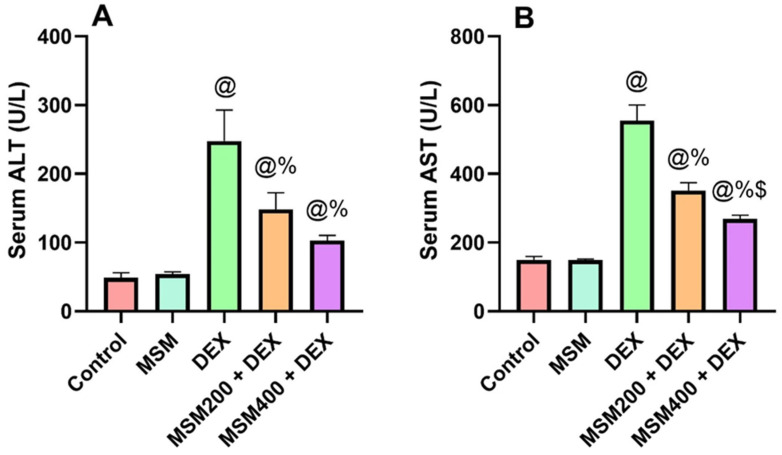
**Impact of MSM (200 and 400 mg/kg) on liver function.** (**A**) Serum ALT level, (**B**) Serum AST level. Data were expressed as mean ± SD, *n* = 4. @, %, $ *p* < 0.05 compared to the CTR, DEX, MSM200 + DEX, respectively, using one-way ANOVA, followed by the Tukey–Kramer multiple comparisons post hoc test.

The actions of MSM were investigated regarding liver functions and architecture while co-administering DEX. The liver function markers of ALT and AST were evaluated in the extracted serum of rats. The single treatment with DEX prominently elevated the levels of both ALT (by 5-fold) and AST (by 3.7-fold) as compared to the control drug-untreated group. Conversely, the treatment with MSM at doses of 200 and 400 mg/kg evidently diminished the values of ALT (by 40% and 58.3%, respectively) and AST (by 36.6% and 51.5%, respectively) compared to the DEX-treated group. It was also noted that the higher doses of 400 mg/kg have an additional reduction in these markers compared to lower doses of 200 mg/kg. It was also noteworthy that the monotherapy with MSM produced no significant influence on AST and ALT grading in comparison to control treatment, highlighting its beneficial effects (Figure 2).

**Figure 3:**
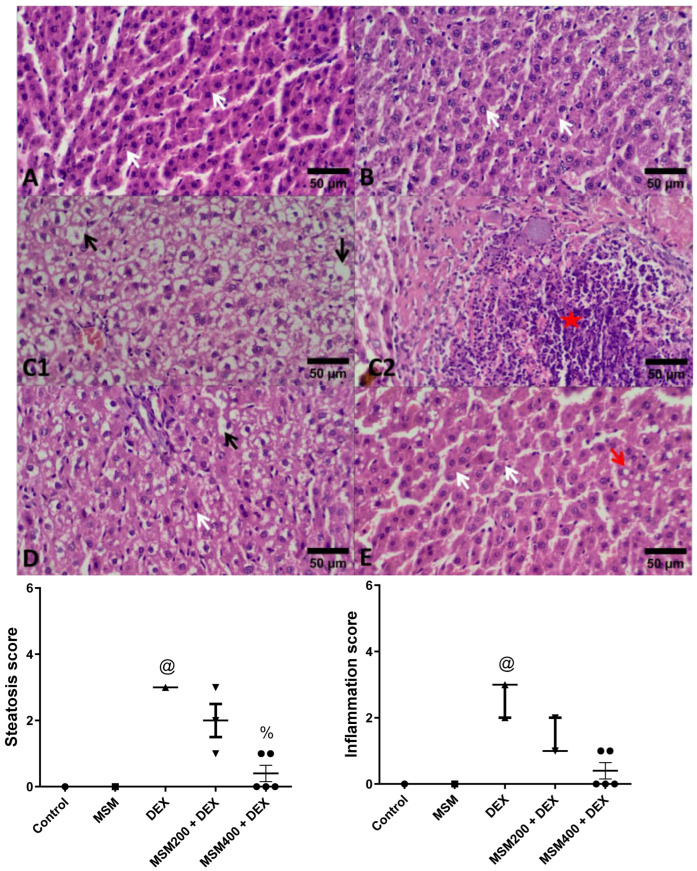
**Impact of MSM (200 and 400 mg/kg) on liver structure alterations, steatosis and inflammation score, showing representative photomicrograph of hepatic tissue from different treatment groups.** (**A**) **Control group,** (**B**) **MSM-only group,** (**C1**,**C2**) **DEX-administered group,** (**D**) **low dose MSM-treated group and** (**E**) **high dose MSM-treated group; the original images can be found in the Supplementary Materials.** White arrows represented organized polygonal hepatocytes, abundant eosinophilic cytoplasm, and central rounded nuclei. Black arrows represented severely diffuse macrovesicular steatosis, where large fat vacuoles displace the cytoplasm and nuclei. Red star represents hepatocyte injury, in the form of necro-inflammatory changes with dense neutrophils and lymphocytes. Red arrow represented a moderate decrease in macrovesicular steatosis with partial restoration of the normal eosinophilic cytoplasm. Shows marked restoration of near-normal hepatic architecture with only a few scattered microvesicular steatosis. Magnification power: ×400. Data are expressed as median ± interquartile range, *n* = 5 and were statistically analyzed using the Kruskal–Wallis test followed by the Dunn multiple comparisons test. @ % *p* < 0.05 compared to the CTR and DEX groups, respectively.

These biochemical results were further confirmed by histopathological observations, which showed normal histological structure of the liver with organized polygonal hepatocytes, abundant eosinophilic cytoplasm, and central rounded nuclei in both the control group and MSM only group Dex-administered group showed severe diffuse macrovesicular steatosis, where large fat vacuoles displace the cytoplasm and nuclei and hepatocyte injury, in the form of necro-inflammatory changes with dense neutrophils and lymphocytes. The low-dose MSM-treated group revealed a moderate diminution in macrovesicular steatosis with fair restoration of the normal eosinophilic cytoplasm. The high-dose MSM-treated group exhibited marked restoration of near-normal hepatic architecture with only a few scattered microvesicular steatosis. The semiquantitative scoring of inflammation and steatosis confirmed the pathological severity with a higher lesion score in the DEX group compared to the control and other treated groups (Figure 3).

**Figure 4:**
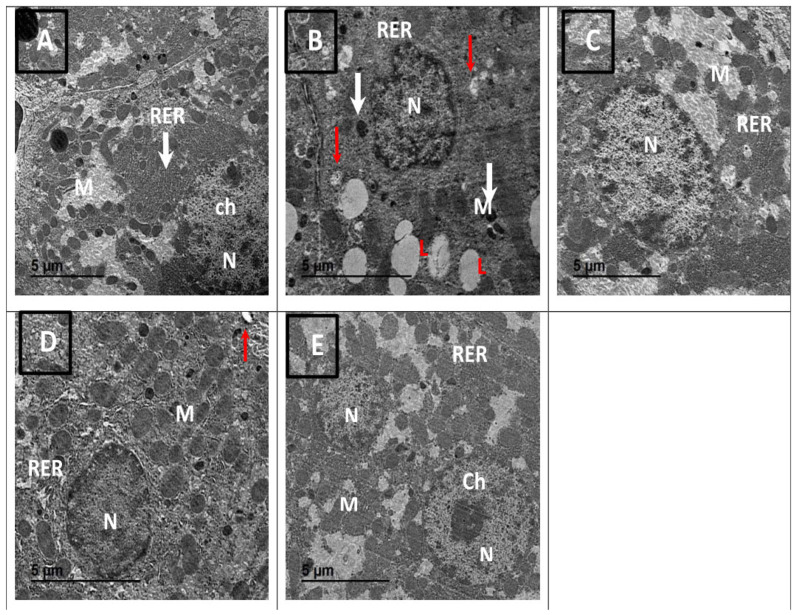
**Impact of MSM (200 and 400 mg/kg) on liver ultrastructure alterations, the original images can be found in the Supplementary Materials.** Transmission electron micrographs of a section in the rat liver of control and experimental groups. (**A**) Control group showed hepatocytes with a normal euchromatic nucleus (N), homogeneously distributed chromatin (ch), and well-preserved mitochondria (M) within the cytoplasm, in addition to normally organized profiles of rough endoplasmic reticulum (RER). (**B**) DEX disease group showed severe ultrastructural changes characterized by disruption of normal hepatocellular architecture, irregularly shaped nuclei (N), and disturbed cellular components. The presence of multiple lipid droplets (L), noted cytoplasm vacuolated (red arrow), degenerated electron-dense mitochondria (M), pyknotic nuclei (N), numerous lysosomes (white arrow), and prominently dilated rough endoplasmic reticulum (RER) was clearly noted. (**C**) MSM400 normal group showed hepatocytes with large, normal, spherical nuclei (N); the cytoplasm contained well-preserved mitochondria and normally arranged rough endoplasmic reticulum. (**D**) MSM200 + DEX group demonstrating hepatocytes with nuclei (N) showing mild peripheral chromatin condensation (ch). The cytoplasm contained abundant mitochondria with normal morphology and variable sizes (red arrows), in addition to areas of dilated rough endoplasmic reticulum (RER). (**E**) MSM400 + DEX group revealed marked improvement of hepatic cellular architecture approaching that of the control group, characterized by an almost normal nucleus (N) and cytoplasm with intact mitochondria (M). CTR: control, DEX: dexamethasone, and MSM: methylsulfonylmethane.

Regarding TEM results, micrographs of rat liver sections from the control group showed hepatocytes with a normal euchromatic nucleus, homogeneously distributed chromatin, and well-preserved mitochondria within the cytoplasm, in addition to normally organized profiles of rough endoplasmic reticulum. The DEX disease group showed severe ultrastructural changes characterized by disruption of normal hepatocellular architecture, irregularly shaped nuclei, and disturbed cellular components. The presence of multiple lipid droplets, noted cytoplasm vacuolated, degenerated electron-dense mitochondria, pyknotic nuclei, numerous lysosomes, and prominently dilated rough endoplasmic reticulum was clearly noted. The MSM (400 mg/kg) group showed hepatocytes with large, normal, spherical nuclei; the cytoplasm contained well-preserved mitochondria and normally arranged rough endoplasmic reticulum. MSM 200 + DEX demonstrated hepatocytes with nuclei showing mild peripheral chromatin condensation. The cytoplasm contained abundant mitochondria with normal morphology and variable sizes, in addition to areas of dilated rough endoplasmic reticulum. MSM400 + DEX group revealing marked improvement of hepatic cellular architecture approaching that of the control group, characterized by an almost normal nucleus and cytoplasm with intact mitochondria (Figure 4).

### 3.3. Impact of MSM (200 and 400 mg/kg) on Lipid Profile

**Figure 5:**
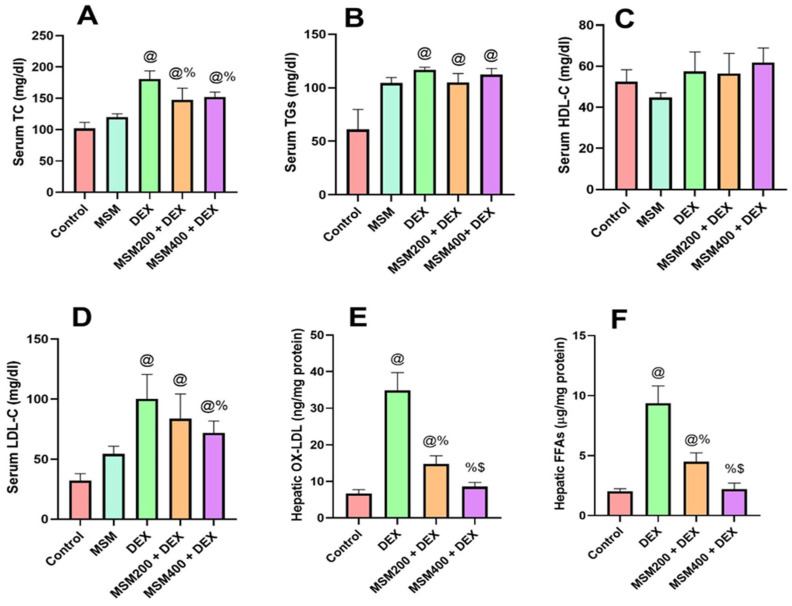
**Impact of MSM (200 and 400 mg/kg) on lipid profile. (A) Serum TC, (B) Serum TGs, (C) Serum HDL-C, (D) Serum LDL-C, (E) Hepatic OX-LDL, (F) Hepatic FFAs.** Data were expressed as mean ± SD, *n* = 4–6. @, %, $ *p* < 0.05 compared to the Control, DEX, MSM200 + DEX, respectively, using one-way ANOVA followed by the Tukey–Kramer multiple comparisons post hoc test. TC: total cholesterol, DEX: dexamethasone, FFAs: free fatty acids, HDL-C: high-density lipoprotein, LDL-C: low-density lipoprotein, MSM: methylsulfonylmethane, OX-LDL: oxidized low-density lipoprotein and TGs: triglycerides.

Hyperlipidemia is commonly observed with DEX application. Hence, we decided to measure the indicators of lipid contents after monotherapy of DEX and its combination with MSM used in two incremental doses. The results showed that DEX prominently raised levels of TC (by 1.7-fold), TGs (by 1.9-fold), LDL-C (by 3.1-fold), OX-LDL (by 5.2-fold) and FFA (by 4.5-fold) but did not affect HDL-C (Figure 5A–F). However, MSM administration (200 and 400 mg/kg) successfully regressed the dyslipidemic changes induced by DEX treatment including reduction in levels of TC (by 18.3% and 16%, respectively), TGs (by 10% and 3.7%, respectively), LDL-C (by 16.4% and 28.1%, respectively), OX-LDL (by 57.6% and 75.3%, respectively) and FFA (by 51.9% and 76.4%, respectively). The antilipidemic actions of MSM were well demonstrated against the hyperlipidemia induced by DEX.

### 3.4. Impact of MSM (200 and 400 mg/kg) on SGK1:

**Figure 6:**
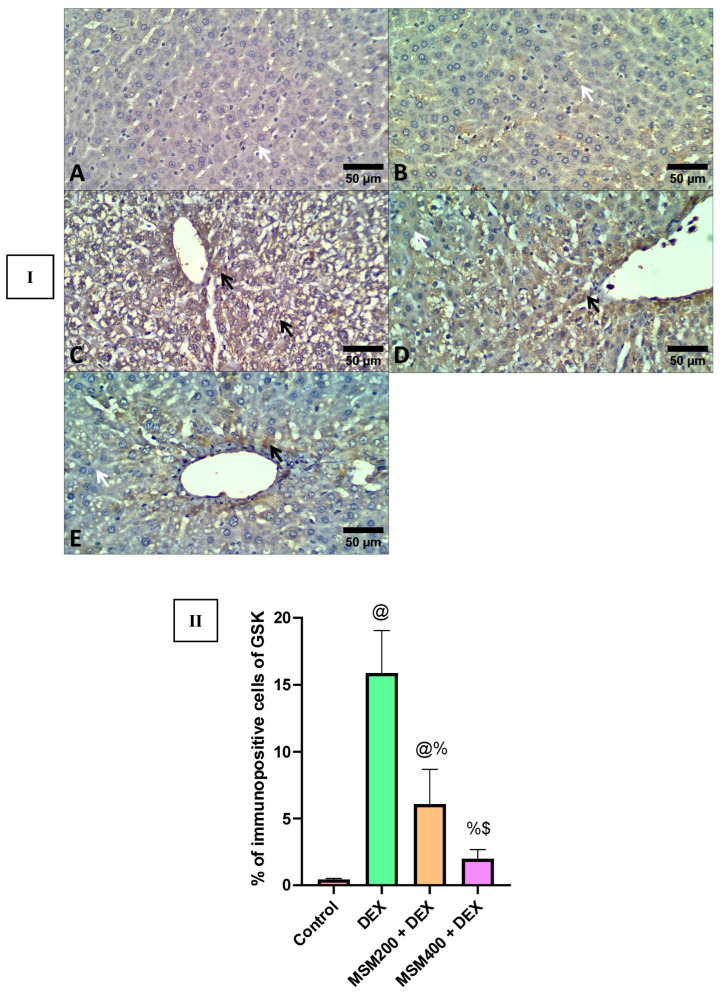
**Immunohistochemical staining and image analysis of SGK1. (I) Immunostained hepatic sections against SGK1.** (**A**) **Control group,** (**B**) **MSM-only group,** (**C**) **DEX-administered group,** (**D**) **low dose MSM-treated group and** (**E**) **high dose MSM-treated group; the original images can be found in the Supplementary Materials. (II) % of immunopositive cells of sgk1.** Magnification power: ×400. White arrows: hepatocytes appear with clear, blue-stained nuclei and pale cytoplasm. black arrows: strong and diffuse positive brown immunoexpression. Magnification power: ×400. Data were expressed as mean ± SD, *n* = 5. @, %, $ *p* < 0.05 compared to the Control, DEX, MSM200 + DEX, respectively, using one-way ANOVA followed by the Tukey–Kramer multiple comparisons post hoc test. DEX: dexamethasone, MSM: methylsulfonylmethane and SGK1: serum glucocorticoid kinase 1.

Figure 6 concerning IHC staining of SGK1 revealed that the control group and MSM-only group exhibited negative immunoexpression in hepatocytes. The faint brownish tint observed is considered non-specific background staining, while the hepatocytes maintain clear, blue-stained nuclei (white arrows). The DEX-administered group demonstrated a strong and diffuse positive brown immunoexpression, showing a granular cytoplasmic pattern across the hepatic lobules. The low dose MSM-treated group exhibited a moderate and focal positive expression. True brown positivity is localized in specific areas, especially around the central vein, while other areas show restoration of negative expression despite the presence of a non-specific background tint. The high dose MSM-treated group revealed a marked reduction in SGK1 positivity. Most hepatocytes are negative, with only very few cells near the central vein displaying faint cytoplasmic staining. This was quantified by a noteworthy increase in % immunopositive cells in DEX competed with the control group. MSM treatment (200 mg/kg) demonstrated a moderate lessening in % of immunopositive cells of SGK1 compared to the DEX group. Pretreatment with MSM (400 mg/kg) revealed a meaningful decrease in % of immunopositive cells of SGK1 as contrasted to the DEX group.

### 3.5. Impact of MSM (200 and 400 mg/kg) on AKT/JNK/IRS-1/GLUT4 Pathway

**Figure 7:**
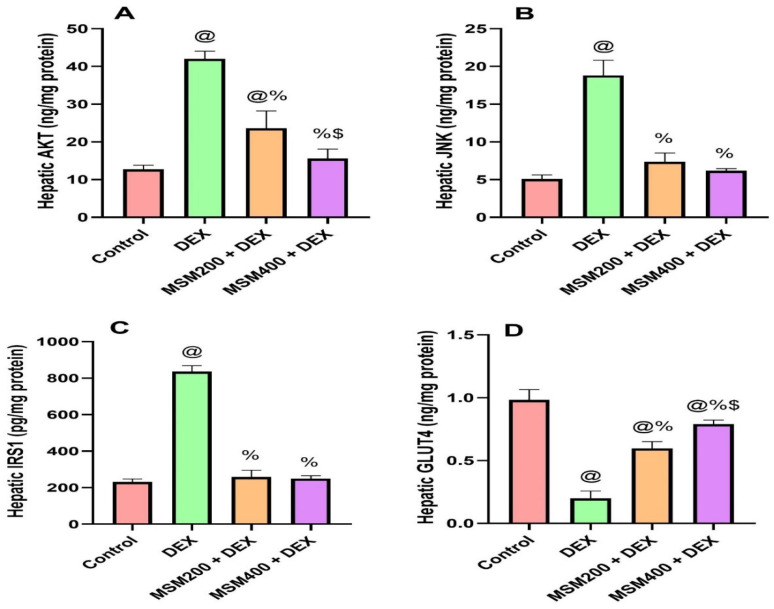
**Impact of MSM (200 and 400 mg/kg) on AKT/JNK/IRS-1/GLUT4 pathway. (A) Hepatic AKT, (B) Hepatic JNK, (C) Hepatic IRS1, (D) Hepatic GLUT4.** Data were expressed as mean ± SD, *n* = 4. @, %, $ *p* < 0.05 compared to the DEX, MSM200 + DEX, respectively, using one-way ANOVA, followed by the Tukey–Kramer multiple comparisons post hoc test. AKT: protein kinase B, CTR: control, DEX: dexamethasone, GLUT4: glucose transporter type 4, IRS-1: insulin receptor substrate-1, JNK: Jun N-terminal kinase and MSM: methylsulfonylmethane.

The underlying metabolic procedures based on the AKT pathway progressing downstream to JNK/IRS-1/GLUT4 was examined. It was noticed that DEX prominently enhanced AKT expression (by 3.3-fold) in the liver tissues, while MSM 200 and 400 mg/kg reduced expression levels (by 43.75% and 62.8%, respectively) (Figure 7A–D). Similarly, JNK level, which functions downstream of AKT, was strikingly upregulated by DEX (by 3.7-fold), while effectively downregulated by MSM (200 and 400 mg/kg) by 60.8% and 67.1%, respectively (Figure 5B). Next, the DEX group exhibited elevated expression of IRS-1 (by 3.6-fold), which was clearly reduced (by 69.1% and 70.1%, respectively) by MSM (200 and 400 mg/kg) administration (Figure 5C). The evaluation of IRS-1 expression impelled us to investigate the status of GLUT4 in liver tissues on treatment with DEX and MSM. The results manifested that DEX inhibited the GLUT4 levels (by 79.6%) compared to the control group, while MSM (200 and 400 mg/kg) led to recovery of GLUT4 expression status (by 2.9- and 3.9-fold, respectively) (Figure 5D). It was also showed that the restoration of GLUT4 was more discernible at a higher dose of MSM than at a lower dose. This highlighted that MSM has a very conspicuous effect on this glucose transporter.

### 3.6. Impact of MSM (200 and 400 mg/kg) on mTOR and AMPK

**Figure 8:**
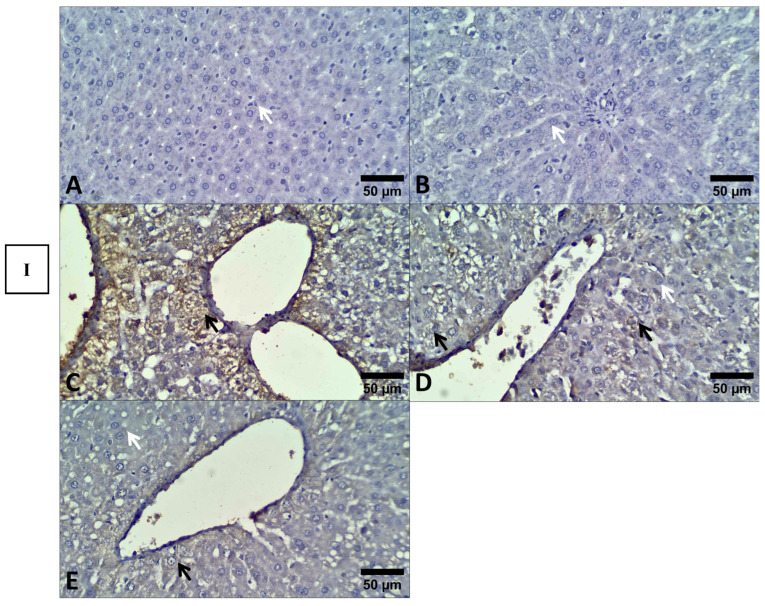

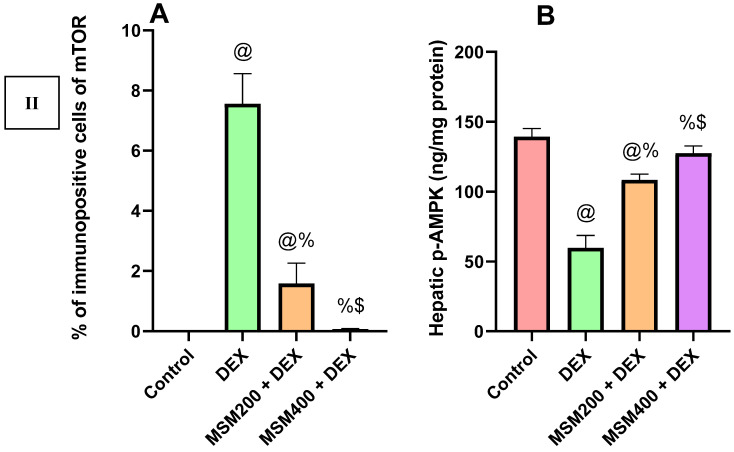
**Impact of MSM (200 and 400 mg/kg) on mTOR and p-AMAPK; the original images can be found in the Supplementary Materials. (I) Immunostained hepatic sections against mTOR.** (**A**) **Control group,** (**B**) **MSM-only group,** (**C**) **DEX-administered group,** (**D**) **low dose MSM-treated group and** (**E**) **high dose MSM-treated group. (II)** (**A**) **% of immunopositive cells of mTOR,** (**B**) **Hepatic p-AMPK.** White arrows: hepatocytes appear with clear, blue-stained nuclei and pale cytoplasm. Black arrows: strong and diffuse positive brown immunoexpression. Magnification power: ×400. Data were expressed as mean ± SD, *n* = 4–5. @, %, $ *p* < 0.05 compared to the DEX, MSM200 + DEX, respectively, using one-way ANOVA, followed by the Tukey–Kramer multiple comparisons post hoc test. p-AMPK: phosphorylated AMP-activated protein kinase, DEX: dexamethasone, MSM: methylsulfonylmethane and mTOR: mechanistic target of Rapamycin.

IHC staining of mTOR revealed that both the control group and MSM-only group exhibited a normal histological pattern with negative to very faint cytoplasmic expression. The hepatocytes maintain their regular polyhedral shape with prominent blue nuclei. The DEX-administered group revealed a strong and extensive positive brown cytoplasmic expression, mainly perivascular distribution. The staining is particularly intense in the areas exhibiting macrovesicular steatosis. The low dose MSM-treated group exhibited a moderate reduction in immunoexpression, while some cells near the central vein still showed positive brown staining, and other areas showed clear restoration of the negative staining pattern. The high dose MSM-treated group demonstrated a marked decrease in mTOR positivity. Most hepatocytes display a negative expression, with only minimal, faint staining remaining in a few cells (Figure 8).

After investigating AKT and related downstream components, we opted to ascertain whether AKT affects AMPK expression. It was demonstrated that DEX application vividly diminished AMPK levels in rats (Figure 8). Conversely, compared to the control group, MSM elevated AMPK expression, particularly at higher doses of 400 mg/kg. There was also a significant difference in levels of expression of AMPK between higher and lower doses of MSM. These results show the dose-dependent restoration of AMPK/mTOR signaling by MSM treatments. This AMPK/mTOR restoration contributes to the regulation of glucose metabolism (Figure 8).

### 3.7. Impact of MSM (200 and 400 mg/kg) on ERK/PI3K/FOXO3/PPARγ Pathway

**Figure 9:**
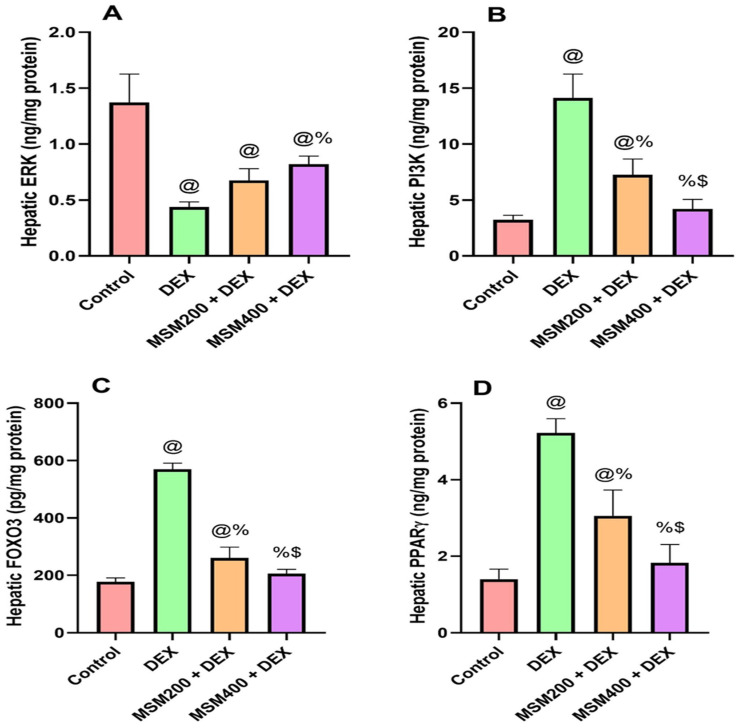
**Impact of MSM (200 and 400 mg/kg) on ERK/PI3K/FOXO3/PPARγ pathway.** (**A**) Hepatic ERK, (**B**) Hepatic PI3K, (**C**) Hepatic FOXO3, (**D**) Hepatic **PPARγ**. Data were expressed as mean ± SD, *n* = 4. @, %, $ *p* < 0.05 compared to the DEX, MSM200 + DEX, respectively, using one-way ANOVA, followed by the Tukey–Kramer multiple comparisons post hoc test. DEX: dexamethasone, ERK: extracellular signal-regulated kinase, FOXO3: forkhead box O3, MSM: methylsulfonylmethane, PI3K: phosphoinositide 3-kinase and PPARγ: peroxisome proliferator-activated receptor gamma.

To investigate the broader functions of DEX-induced metabolic derangements and effective management by MSM, we examined the ERK molecule and associated components. The ERK level was considerably reduced (by 68%) in the hepatic tissues of rats given DEX treatment compared to the vehicle normal group (Figure 9A). In contrast, compared to the DEX-injected group, the MSM at 200 and 400 mg/kg markedly raised ERK levels (by 1.5- and 1.8-fold, respectively). Similarly, PI3K levels were evidently greater (by 4.3-fold) in the DEX-administered group relative to the normal control group. Meanwhile, MSM in both doses drastically regressed PI3K levels (by 48.6% and 70.2%, respectively) (Figure 9B). Additionally, compared to a lower dose of 200 mg/kg, the higher dose of 400 mg/kg showed a significantly greater reduction.

Next, when the status of FOXO3 was quantified in the murine hepatic tissues of the DEX-treated group, it was revealed that DEX significantly raised FOXO3 levels (by 3.2-fold) relative to the control murine group (Figure 9C). Nevertheless, the levels of FOXO3 were profoundly decreased (by 54.3% and 63.9%, respectively) following administration of MSM at both incremental dosages. Moreover, DEX drastically increased PPARγ expression (by 3.7-fold) in comparison to the control group (9D). However, MSM effectively reduced PPARγ values at both doses (200 and 400 mg/kg) by 41.5% and 65.1%, respectively, when compared to the DEX-administered group. Furthermore, compared to the 200 mg/kg dose, the 400 mg/kg dose manifested an evidently greater reduction. Hence, it was discovered that MSM efficiently modulated the ERK/PI3K/FOXO3/PPARγ axis.

### 3.8. Impact of MSM (200 and 400 mg/kg) on SREBP-1/GSK/GS/PEPCK Pathway

**Figure 10:**
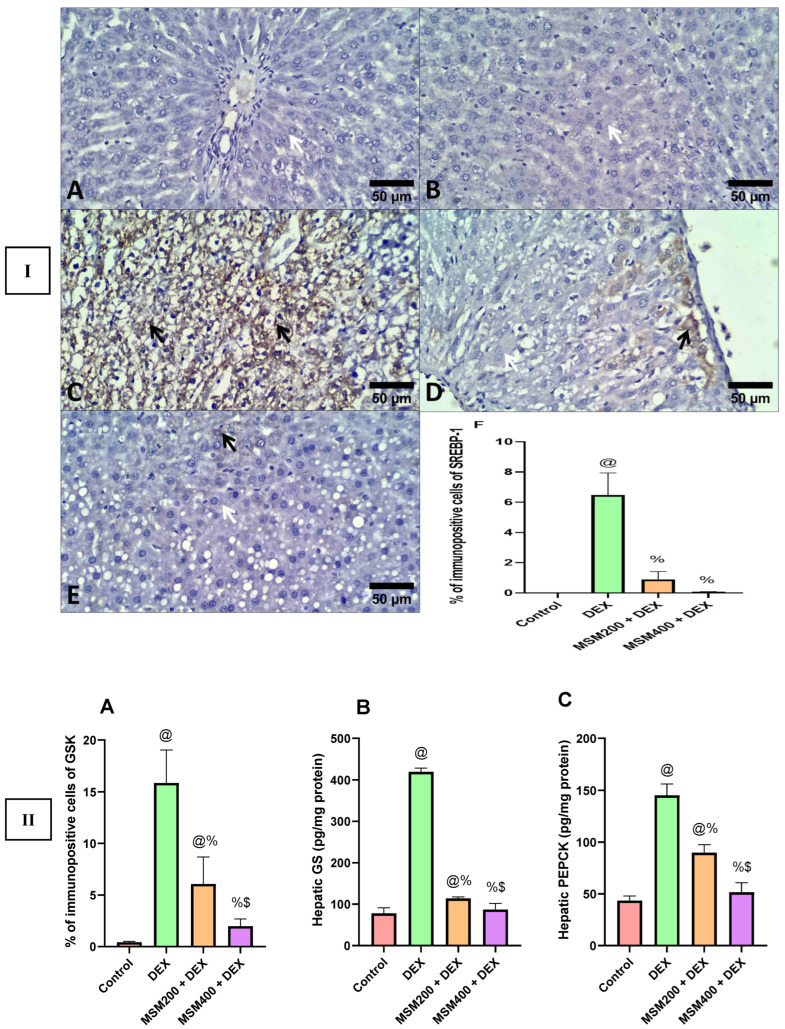
**Impact of MSM (200 and 400 mg/kg) on SREBP-1/GSK/GS/PEPCK pathway. (I)** IHC staining of SREBP1 showed (**A**) control group, (**B**) MSM-only group, (**C**) DEX group, (**D**) MSM200 + DEX, (**E**) MSM400 + DEX and (**F**) % of immunopositive cells of SREBP-1; the original images can be found in the Supplementary Materials. **(II)** (**A**) **Hepatic GSK,** (**B**) **Hepatic GS,** (**C**) **Hepatic PECK.** White arrows: hepatocytes appear with clear, blue-stained nuclei and pale cytoplasm. Black arrow, strong and diffuse positive brown immunoexpression. Data were expressed as mean ± SD, *n* = 4–5. @, %, $ *p* < 0.05 compared to the DEX, MSM200 + DEX, respectively, using one-way ANOVA, followed by the Tukey–Kramer multiple comparisons post hoc test. DEX: dexamethasone, GS: glycogen synthase, GSK: glycogen synthase kinase, MSM: methylsulfonylmethane and PEPCK: phosphoenolpyruvate carboxykinase.

IHC staining of SREBP1 showed control group and MSM-only group exhibited normal hepatic lobular architecture with negative to negligible immunoexpression. The hepatocytes appear with clear, blue-stained nuclei and pale cytoplasm. The DEX-administered group revealed a strong and diffuse positive brown immunoexpression. The staining is prominently localized in both the cytoplasm and nuclei of hepatocytes surrounding the large macrovesicular fat vacuoles. The low dose MSM-treated group illustrated a moderate and localized positive expression. A noticeable reduction in the intensity and distribution of the brown stain is observed compared to the Dexa group, although some areas still show residual activity. The high dose MSM-treated group demonstrated a marked reduction in expression. Most hepatocytes showed weak to negative staining, with only occasional cells showing faint cytoplasmic positivity (Figure 10).

The investigation of hepatic levels of GSK, GS and PEPCK demonstrated a considerably greater expression (by 4.1-, 5.3- and 3.3-fold, respectively) in the DEX-administered group than the control vehicle-treated group. However, MSM application led to an evident dose-incremental diminution of expression of GSK (by 37.6% and 70.8%, respectively), GS (by 72.8% and 79.2%, respectively) and PEPCK (by 38.1% and 64.4%, respectively) in contrast to the DEX-treated group (Figure 10).

### 3.9. Impact of MSM (200 and 400 mg/kg) on NLRP3, NF-κB and IL-6 Levels

**Figure 11:**
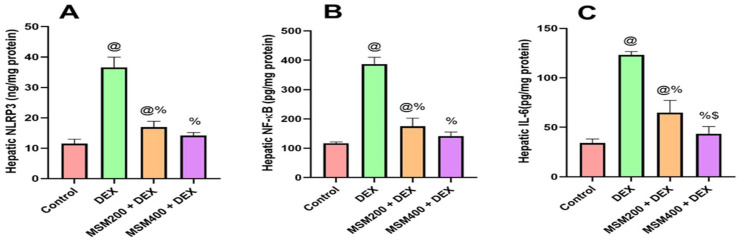
**Impact of MSM (200 and 400 mg/kg) on NLRP3, NF-κB and IL-6 levels.** (**A**) Hepatic NLRP3, (**B**) Hepatic **NF-κB**, (**C**) **Hepatic IL-6**. Data were expressed as mean ± SD, *n* = 4. @, %, $ *p* < 0.05 compared to the DEX, MSM200 + DEX, respectively, using one-way ANOVA, followed by the Tukey–Kramer multiple comparisons post hoc test. DEX: dexamethasone, IL-6: interleukin-6, MSM: methylsulfonylmethane, NF-κB: nuclear factor kappa-light-chain-enhancer of activated B cells, and NLRP3: nucleotide-binding oligomerization domain, Leucine-rich repeat and Pyrin domain-containing 3.

To determine the underlying inflammatory component, we quantified the expression status of inflammatory markers, such as NLRP3, NF-κB and IL-6. The results depicted that DEX significantly enhanced the formation of NLRP3 (by 3.1-fold), NF-κB (by 3.3-fold) and IL-6 (by 3.6-fold), highlighting the induction of inflammation by this steroidal agent. However, after treatment with MSM 200 and 400 mg/kg, it was evident that there was downregulation in a dose-dependent fashion in levels of NLRP3 (by 53.6% and 61.1%, respectively), NF-κB (by 54.7% and 63.3%, respectively) and IL-6 (by 47.3% and 64.6%, respectively) (Figure 11A–C). Hence, these results indicated that DEX induced an inflammatory process in rats through upregulation of NLRP3, NF-κB and IL-6; however, MSM exerted anti-inflammatory actions by depressing the levels of both inflammatory mediators. Furthermore, the higher dosage of MSM produced more distinct effects than the lower dosage of the drug.

### 3.10. Impact of MSM (200 and 400 mg/kg) on Oxidative Stress

**Figure 12:**
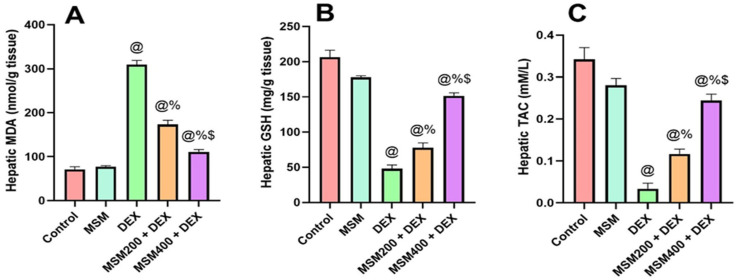
**Impact of MSM (200 and 400 mg/kg) on oxidative stress.** (**A**) Hepatic MDA, (**B**) Hepatic GSH, (**C**) Hepatic TAC. Data were expressed as mean ± SD, *n* = 4–6. @, %, $ *p* < 0.05 compared to the control, DEX, MSM200 + DEX, respectively, using one-way ANOVA, followed by the Tukey–Kramer multiple comparisons post hoc test. DEX: dexamethasone, GSH: glutathione, MDA: malondialdehyde, MSM: methylsulfonylmethane and TAC: total antioxidant capacity.

To further elucidate the underlying role of oxidative stress in hyperglycemic disorders, we explored MDA, GSH, and TAC levels. DEX was found to elevate MDA levels (by 4.3-fold) while concurrently reducing GSH formation (by 76.7%) compared to the control group (Figure 12A,B). The statistical analysis also revealed that the TAC in the DEX group was quite diminished (by 90.2%) as compared to the control group (Figure 12C). While it was determined that MSM comprehensively retarded the development of oxidative markers in contrast to the DEX group. MDA levels were also significantly reduced (by 44.1% and 64.1%, respectively) by both doses of MSM, indicating very effective antioxidant features of MSM. Thus, through increased status of GSH (by 1.6- and 3.1-fold, respectively) and TAC (by 3.5- and 7.3-fold, respectively), MSM markedly improved the antioxidant levels. Altogether, these results denote the defensive actions of MSM against DEX-induced oxidative harms.

### 3.11. Impact of MSM (200 and 400 mg/kg) on CD34 and VEGF

**Figure 13:**
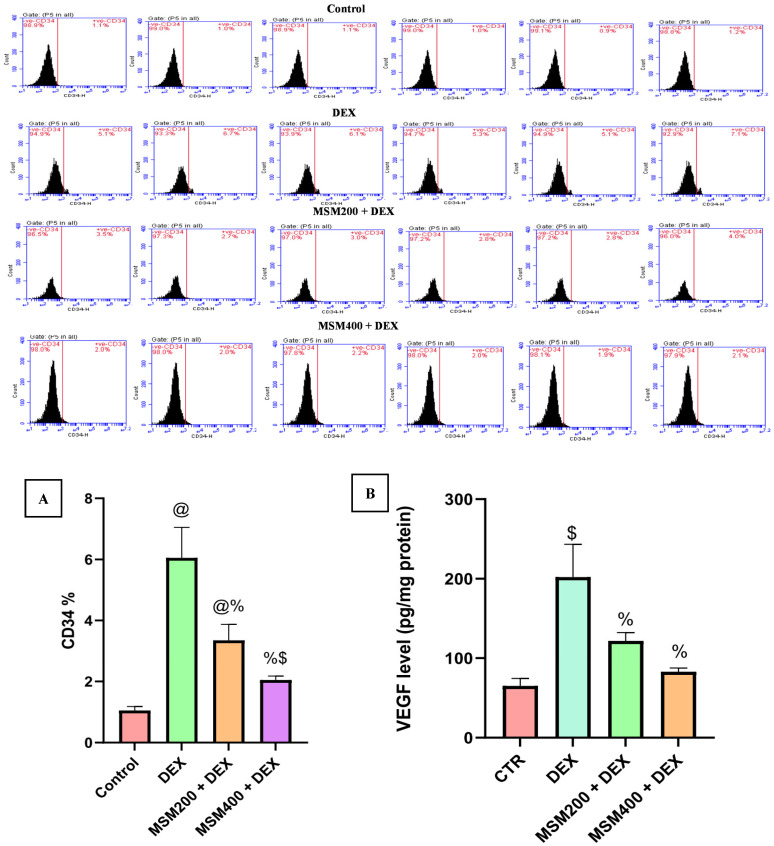
**Effect of MSM on CD34 expression** (flow cytometric analysis) **and VEGF level. (A) Flowcytometry analysis of CD34, (B) Hepatic VEGF.** Data were expressed as mean ± SD, *n* = 4. @, %, $ *p* < 0.05 compared to the control, DEX, MSM200 + DEX, respectively, using one-way ANOVA, followed by the Tukey–Kramer multiple comparisons post hoc test. DEX: dexamethasone, CD34: cluster of differentiation 34 and VEGF: vascular endothelial growth factor.

Flow cytometric analysis demonstrated a significant increase in CD34 expression in the DEX-treated group compared to the control group, indicating enhanced endothelial activation and angiogenic response. Interestingly, treatment with our tested compound markedly attenuated this elevation in a dose-dependent manner, with the higher dose showing a more pronounced reduction in CD34 expression. Similarly, VEGF levels were significantly elevated following DEX administration, whereas treatment with the investigated drug significantly improved VEGF levels, restoring them toward near-normal values in a dose-dependent fashion (Figure 13).

## 4. Discussion

The present study provided integrated mechanistic evidence that MSM markedly attenuates DEX-induced hepatic IR and metabolic dysfunction through coordinated modulation of oxidative stress, inflammation, insulin signaling, metabolic transcriptional control, and, importantly, angiogenic remodeling. The protective efficacy of MSM appears to arise from its ability to target multiple interconnected molecular networks rather than acting through a single pathway.

Chronic glucocorticoid exposure is strongly associated with systemic IR secondary to metabolic reprogramming and inflammatory recruitment [28]. In the current model, DEX administration significantly upregulated hepatic NLRP3, NF-κB p65, and IL-6 expression, confirming the establishment of a pro-inflammatory hepatic microenvironment. Activation of the NLRP3 inflammasome constitutes a pivotal molecular bridge linking oxidative stress to cytokine amplification and insulin signaling disruption. The marked suppression of NLRP3/NF-κB/IL-6 signaling by MSM in a dose-dependent manner aligns with previous reports demonstrating MSM-mediated inhibition of inflammatory cytokine production [19,29] and inflammasome modulation [30]. These findings reinforce the concept that MSM exerts upstream anti-inflammatory control rather than merely dampening terminal cytokine output.

Oxidative stress represents a central upstream driver in this inflammatory cascade. DEX-treated animals exhibited elevated MDA levels alongside depleted GSH and TAC, indicating profound redox imbalance [31]. ROS are well-established activators of NLRP3 and NF-κB signaling, thereby amplifying inflammatory transcription. MSM significantly restored antioxidant defenses and reduced lipid peroxidation, confirming its potent free radical scavenging capacity. As a naturally occurring organosulfur compound, MSM contributes sulfur for glutathione synthesis and enhances endogenous redox buffering. This antioxidant restoration likely constitutes the primary initiating event through which downstream inflammatory and metabolic pathways are normalized.

The insulin signaling findings warrant careful interpretation. The paradoxical increase in AKT and IRS-1 levels in DEX-treated rats, which contradicts other reports of glucocorticoid-mediated inhibition of the IRS-1/PI3K/AKT pathway [32], is a particularly significant finding in our current study. Acute DEX therapy was shown by Qi and associates to interrupt insulin signaling in heart tissue. An in-depth assessment of the mechanisms underlying insulin resistance suggested that DEX suppresses Akt phosphorylation and hinders IRS/PI3K/Akt signal transduction in C2C12 myotubes. Hence, there is a need for a critical evaluation of this finding. The liver may enhance distant insulin signaling components while downstream effectors (particularly GLUT4) remain suppressed to overcome peripheral insulin resistance. This tissue-specific compensatory response is likely explained by our observation of elevated hepatic AKT and IRS-1 expression in DEX-treated rats.

In addition, the present study provided further mechanistic insight through the assessment of SGK1 expression. SGK1 is a well-recognized glucocorticoid-responsive kinase that is transcriptionally upregulated upon glucocorticoid receptor activation and functionally overlaps with the PI3K/AKT pathway. Accumulating evidence suggests that persistent SGK1 activation contributes to insulin resistance, metabolic dysregulation, and inflammatory signaling [33,34]. In the current model, DEX administration markedly increased hepatic SGK1 expression, consistent with its glucocorticoid-driven transcriptional regulation. Notably, MSM treatment significantly reduced SGK1 expression toward near-normal levels. Given that SGK1 can enhance NF-κB activity and promote metabolic stress responses, its suppression by MSM further supports the hypothesis that this compound interferes with glucocorticoid-mediated signaling at both transcriptional and post-receptor levels. The downregulation of SGK1 may therefore represent an additional mechanism through which MSM restores insulin signaling balance and attenuates inflammatory amplification.

Energy sensing through the p-AMPK/mTOR axis was markedly disrupted following DEX administration. Given that hyperglycemia, hyperinsulinemia, and lipid accumulation would normally stimulate p-AMPK activation, its suppression reflects maladaptive metabolic signaling. MSM restored p-AMPK activity and corrected mTOR dysregulation, favoring improved fatty acid oxidation and mitochondrial efficiency [16]. This reinforces MSM’s role as a metabolic stabilizer under glucocorticoid stress.

At the transcriptional level, DEX enhanced FOXO3, PPARγ, PEPCK, and GSK-3β expression while suppressing GS, consistent with glucocorticoid-driven gluconeogenesis and impaired glycogen storage. MSM effectively normalized these alterations, particularly suppressing PEPCK expression, suggesting possible modulation of glucocorticoid receptor-dependent transcriptional activity or its co-regulatory complexes. Regulation of the ERK/PI3K/FOXO3/PPARγ crosstalk further indicated that MSM influenced higher-order transcriptional integration nodes controlling metabolic homeostasis.

A novel and particularly significant contribution of the present study lies in the demonstration that DEX-induced hepatic injury is associated with enhanced angiogenic signaling and sinusoidal remodeling. Elevated ROS and NF-κB activation are known to transcriptionally upregulate VEGF, thereby linking oxidative stress and inflammation to angiogenesis. Consistent with this axis, VEGF levels were markedly increased in DEX-treated rats. Concomitantly, CD34 expression was significantly elevated, reflecting endothelial activation and sinusoidal capillarization. Sinusoidal remodeling disrupts hepatocyte blood exchange, promotes hypoxic signaling, and perpetuates insulin resistance and fibrogenic progression. The simultaneous elevation of VEGF and CD34, therefore, indicated that DEX-induced metabolic dysfunction extends beyond intracellular signaling to microvascular structural alterations. Importantly, MSM significantly reduced both VEGF and CD34 expression in a dose-dependent manner. These findings reveal a previously unrecognized anti-angiogenic and endothelial-stabilizing property of MSM. To the best of our knowledge, this is the first study to assess the effect of MSM on hepatic VEGF and CD34 expression. By attenuating ROS accumulation, suppressing NF-κB activation, and reducing VEGF-driven endothelial activation, MSM appears to interrupt the ROS → NLRP3/NF-κB → VEGF → CD34 → sinusoidal remodeling cascade at multiple hierarchical levels.

From a translational standpoint, MSM possessed a distinct advantage due to its safety profile. As a naturally occurring sulfur-containing compound widely used as a dietary supplement, MSM demonstrated high tolerability and minimal toxicity even at relatively elevated doses. This safety margin enhances its potential applicability for long-term management of glucocorticoid-associated metabolic complications.

## 5. Conclusions

Collectively, the present findings demonstrate that MSM effectively mitigates DEX-induced hepatic insulin resistance and metabolic dysfunction through integrated antioxidant, anti-inflammatory, metabolic, and anti-angiogenic mechanisms. In addition to suppressing the NLRP3/NF-κB axis and restoring p-AMPK/mTOR and IRS-1/PI3K/AKT signaling, MSM significantly downregulated glucocorticoid-induced SGK1 expression. By interrupting the redox–inflammatory–angiogenic cascade, including VEGF and CD34-mediated sinusoidal remodeling, and restoring signaling homeostasis at multiple regulatory nodes, MSM emerges as a safe, natural, and mechanistically versatile therapeutic candidate for glucocorticoid-associated metabolic disorders pending further clinical studies.

## 6. Limitations and Future Directions

Despite the comprehensive molecular characterization, certain limitations should be acknowledged. First, direct functional assessment of systemic insulin sensitivity using gold-standard methods such as hyperinsulinemic–euglycemic clamp was not performed. Second, angiogenic remodeling was evaluated through VEGF and CD34 expression without quantitative histomorphometric analysis of sinusoidal density or ultrastructural assessment of endothelial fenestration. Third, causal pathway validation using selective NLRP3 or NF-κB inhibitors would further strengthen mechanistic confirmation. Fourth, a lack of direct assessment of mTOR phosphorylation levels; thus, further studies using Western blot analysis are needed to verify the role of this signaling pathway.

## Figures and Tables

**Table 1:** Commercially available ELISA kits used.

Marker	Cataloge No.	Company (Country)
Protein kinase B (AKT)	MBS3807575	MyBioSource, San Diego, CA, USA
Phosphorylated AMP-activated protein kinase (p-AMPK)	ER1877	FineTest, Wuhan, China
Extracellular signal-regulated kinase (ERK)	MBS034880	MyBioSource, San Diego, CA, USA
Forkhead box O3 (FOXO3a)	MBS9359523	MyBioSource, San Diego, CA, USA
Free fatty acids (FFA)	MBS733451	MyBioSource, San Diego, CA, USA
Glucose transporter type 4 (GLUT4)	CSB-E13908r	CUSABIO, Wuhan, China
Glycogen synthase (GS)	RE2350R	Reed Biotech, Wuhan, China
Glycogen synthase kinase-3 alpha/beta (GSK-3α/β)	MBS2513979	MyBioSource, San Diego, CA, USA
Interleukin-6 (IL-6)	R6000B	R&D Systems, Minneapolis, MN, USA
Insulin receptor substrate-1 (IRS-1)	MBS2706135	MyBioSource, San Diego, CA, USA
c-Jun N-terminal kinase (JNK)	EK306	ELK Biotechnology, Wuhan, China
Nuclear factor kappa-light-chain-enhancer of activated B cells (NF-κB p65)	MBS7226224	MyBioSource, San Diego, CA, USA
Nucleotide-binding oligomerization domain, Leucine-rich repeat and Pyrin domain-containing 3 (NLRP3)	ER1922	FineTest, Wuhan, China
Oxidized low-density lipoprotein (Ox-LDL)	E-EL-R3034	Elabscience, Houston, TX, USA
Phosphoenolpyruvate carboxykinase (PEPCK)	RAT09161	Assay Genie, Dublin, Ireland
Phosphoinositide 3-kinase (PI3K)	MBS9356979	MyBioSource, San Diego, CA, USA
Peroxisome proliferator-activated receptor gamma (PPAR-γ)	MBS2701257	MyBioSource, San Diego, CA, USA

## Data Availability

The data of the current study is available from the corresponding author on reasonable request due to intellectual property considerations and ongoing collaborative research utilizing the primary dataset.

## Supplementary Materials

The following supporting information can be downloaded at: https://www.mdpi.com/article/10.3390/jox16040121/s1, Figure S1: The original images of Figure 3; Figure S2: The original images of Figure 4; Figure S3: The original images of Figure 6; Figure S4: The original images of Figure 8; Figure S5: The original images of Figure 10.

## Abbreviations

The following abbreviations are used in this manuscript:

AKT	Protein kinase B
ALT	Alanine aminotransferase
p-AMPK	Phosphorylated AMP-activated protein kinase
AST	Aspartate alanine aminotransferase
CAM	Complementary and alternative medicine
CMC	Carboxymethylcellulose
DEX	Dexamethasone
ERK	Extracellular signal-regulated kinase
FFA	Free fatty acid
FOXO3a	Forkhead box O3
GLUT4	Glucose transporter type 4
GS	Glycogen synthase
GSH	Glutathione
GSK-3α/β	Glycogen synthase kinase-3 alpha/beta
HDL-C	High-density lipoprotein cholesterol
HOMA-I	Homeostatic Model Assessment for IR
IHC	Immunohistochemical
IRS-1	Insulin receptor substrate-1
IL-6	Interleukin-6
IR	Insulin resistance
JNK	c-Jun N-terminal kinase
LDL-C	Low-density lipoprotein cholesterol
MAFLD	Early non-alcoholic fatty liver diseases
MDA	Malondialdehyde
MSM	Methylsulfonylmethane
mTOR	Mammalian target of Rapamycin
NAFLD	Metabolism-associated fatty liver disease
NF-κB-p65	Nuclear factor kappa-light-chain-enhancer of activated B cells (NF-κB p65)
NLRP3	Nucleotide-binding oligomerization domain, Leucine-rich repeat and Pyrin domain-containing 3
OGTT	Oral glucose tolerance test
Ox-LDL	Oxidized low-density lipoprotein
PPAR-γ	Peroxisome proliferator-activated receptor gamma
PEPCK	Phosphoenolpyruvate carboxykinase
PI3K	Phosphoinositide 3-kinase
ROS	Reactive oxygen species
SGK1	Serum-regulated glucocorticoids kinase 1
SREBP-1	Sterol Regulatory Element-Binding Protein-1
TAC	Total antioxidant capacity
TC	Total cholesterol
TEM	Transmission electron microscopy
TGs	Triglycerides
