# Supplementary figures and images for: Methylsulfonylmethane Attenuates Dexamethasone-Induced Hepatic Insulin Resistance in Rats: Associations with SGK1, p-AMPK/mTOR, Inflammatory and Angiogenic Markers
Source: J Xenobiot. 2026 Jun 30;16(4):121. doi: 10.3390/jox16040121 (PMC13397917; doi:10.3390/jox16040121)

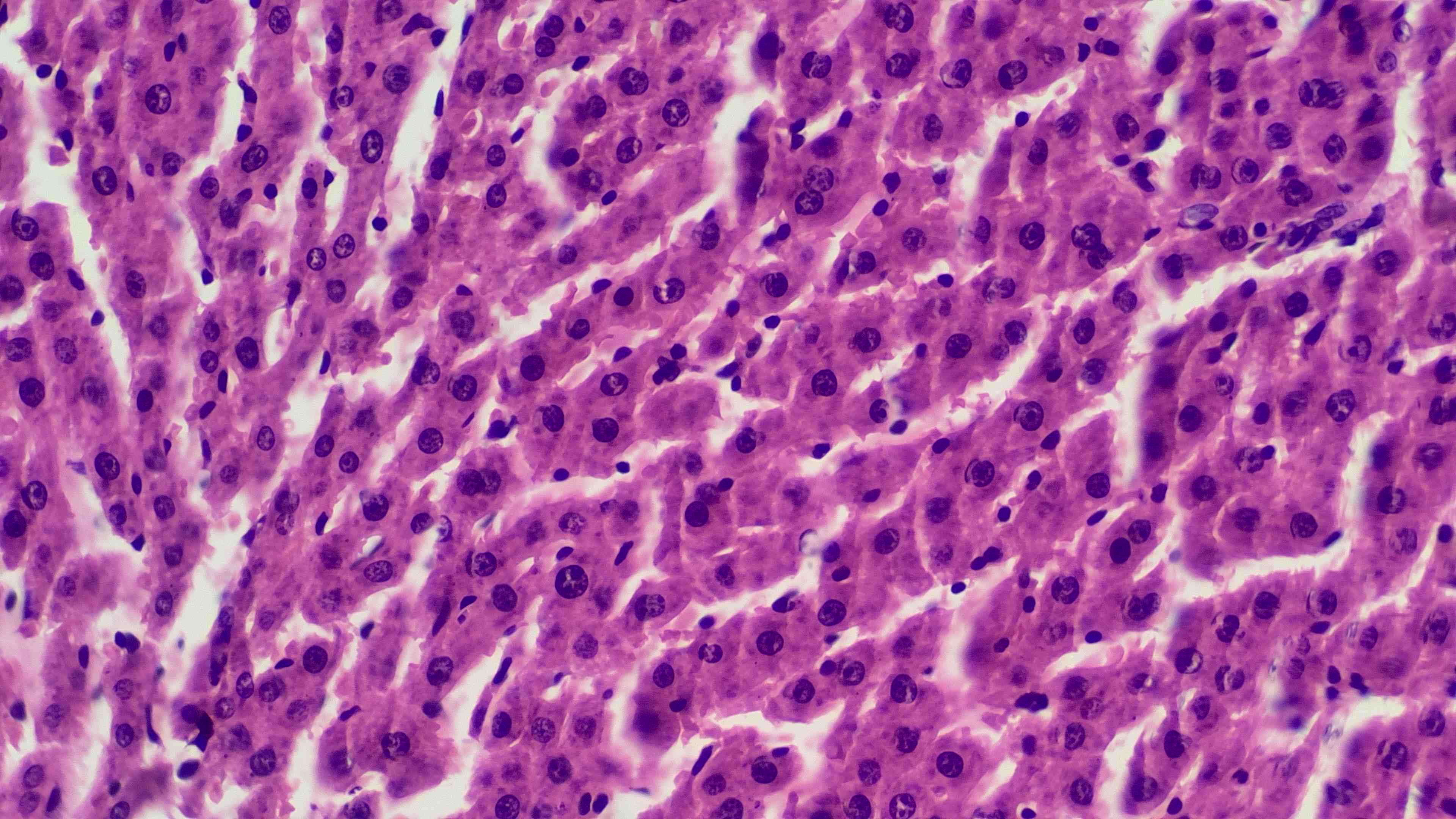

Supplement: Supplementary file 1 [file jox-16-00121-s001.zip › Figure S1 The original images of figure 3/control.JPG]

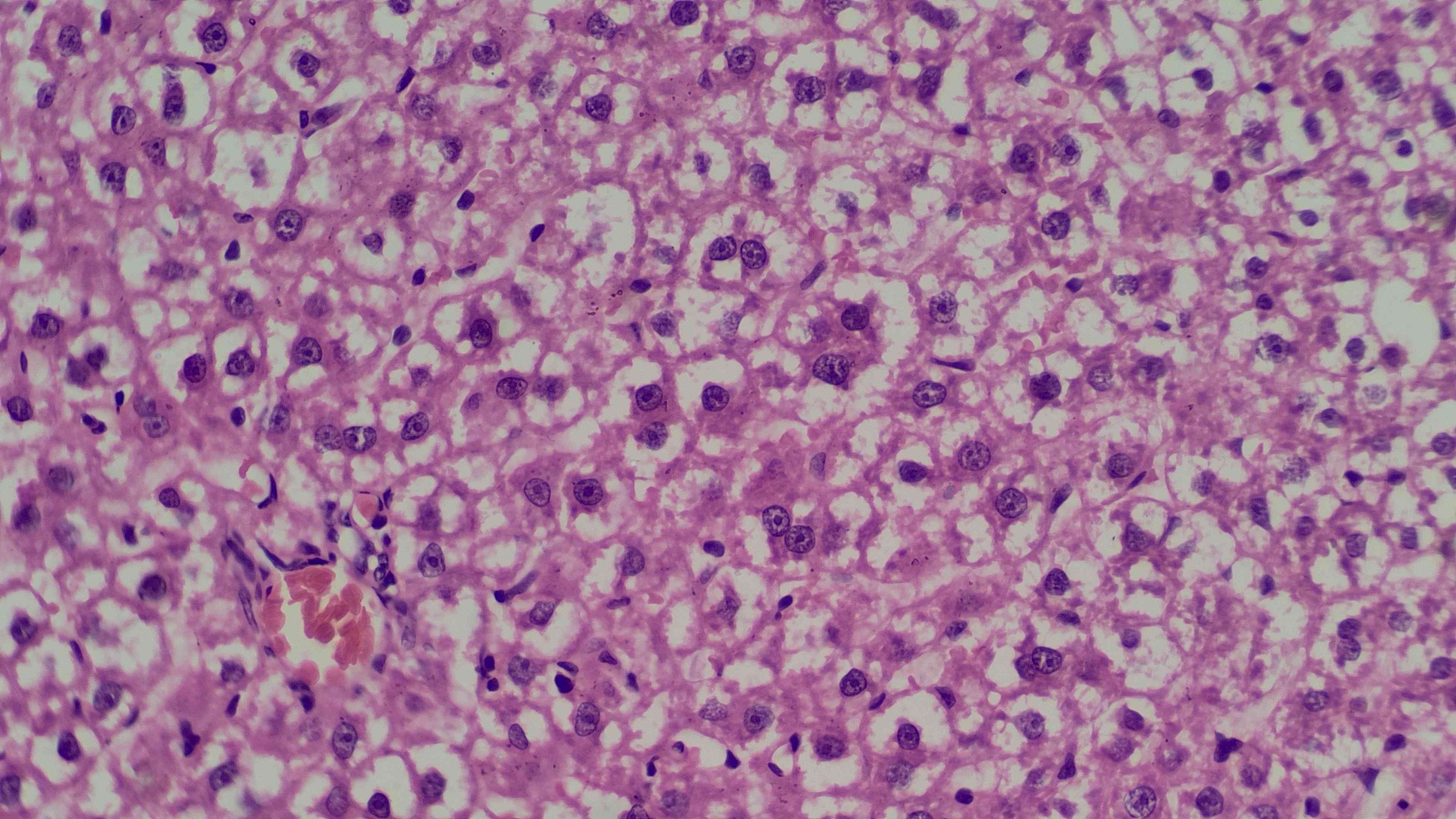

Supplement: Supplementary file 1 [file jox-16-00121-s001.zip › Figure S1 The original images of figure 3/DEXA.JPG]

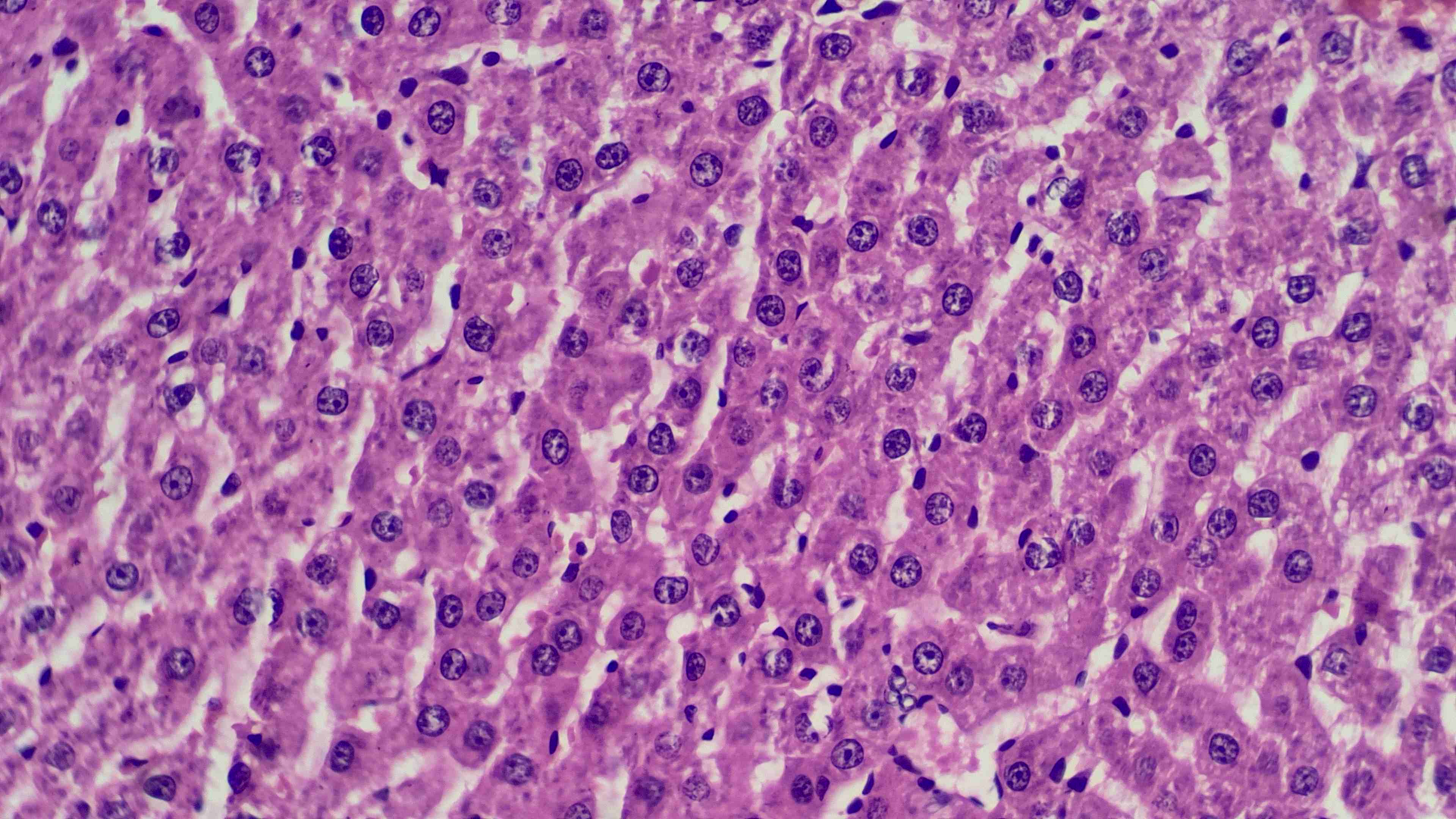

Supplement: Supplementary file 1 [file jox-16-00121-s001.zip › Figure S1 The original images of figure 3/MSM 400.JPG]

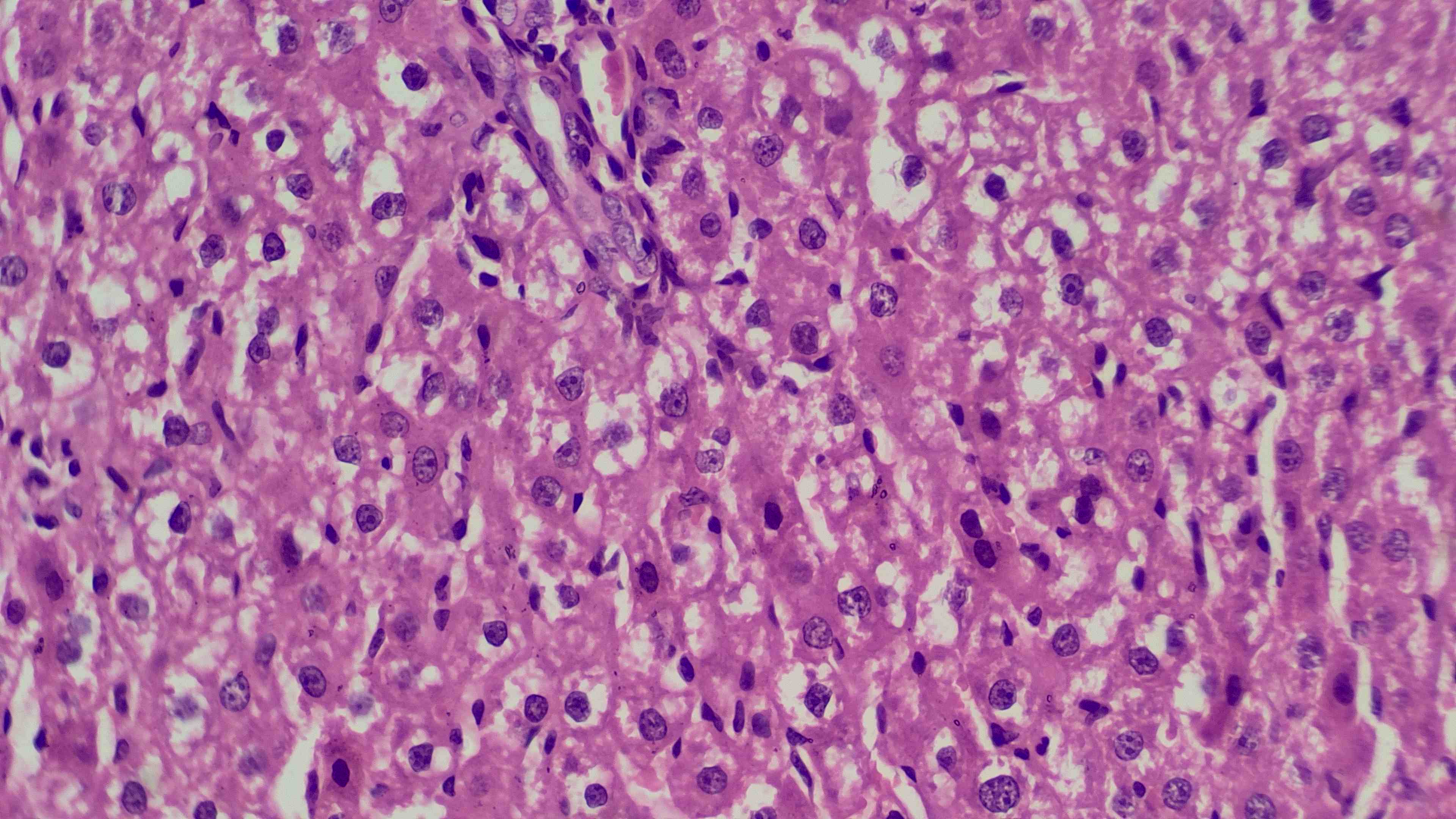

Supplement: Supplementary file 1 [file jox-16-00121-s001.zip › Figure S1 The original images of figure 3/MSM200 + DEXA.JPG]

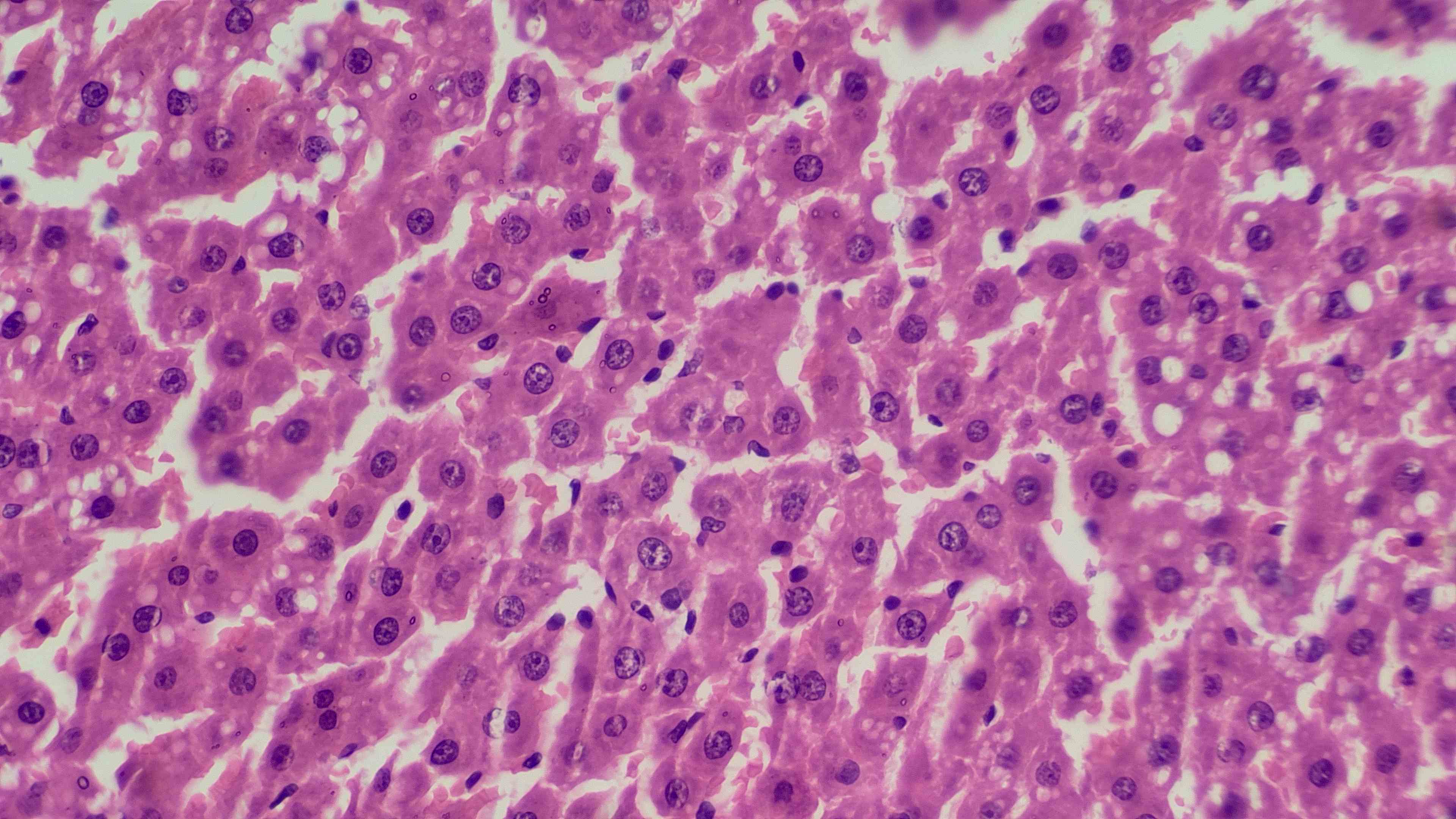

Supplement: Supplementary file 1 [file jox-16-00121-s001.zip › Figure S1 The original images of figure 3/MSM400 + DEXA.JPG]

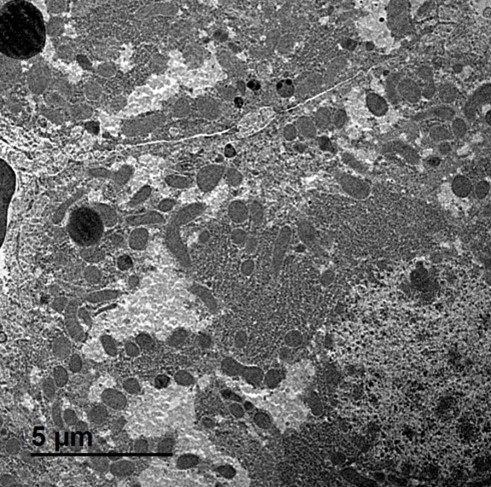

Supplement: Supplementary file 1 [file jox-16-00121-s001.zip › Figure S2 The original images of figure 4/control group.jpg]

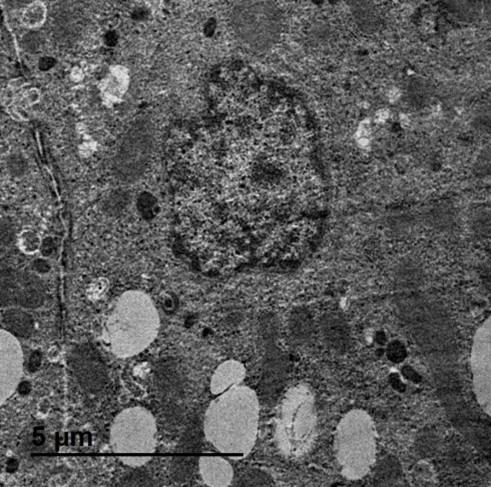

Supplement: Supplementary file 1 [file jox-16-00121-s001.zip › Figure S2 The original images of figure 4/DEXA.jpg]

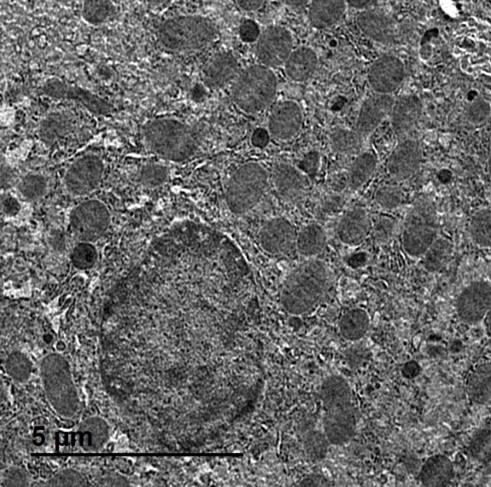

Supplement: Supplementary file 1 [file jox-16-00121-s001.zip › Figure S2 The original images of figure 4/MSM200+DEXA.jpg]

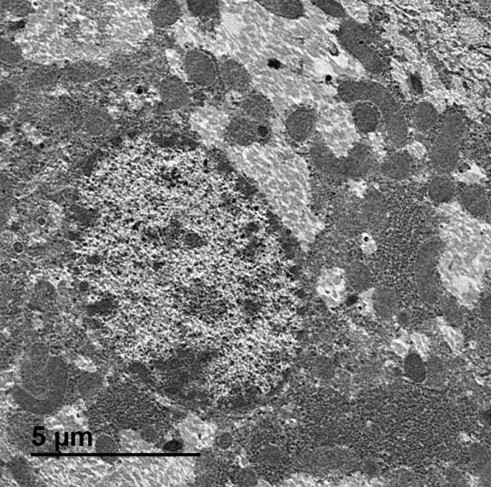

Supplement: Supplementary file 1 [file jox-16-00121-s001.zip › Figure S2 The original images of figure 4/MSM400 control.jpg]

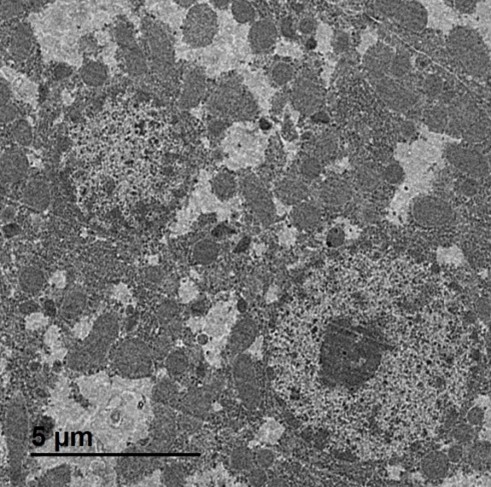

Supplement: Supplementary file 1 [file jox-16-00121-s001.zip › Figure S2 The original images of figure 4/MSM400+DEXA.jpg]

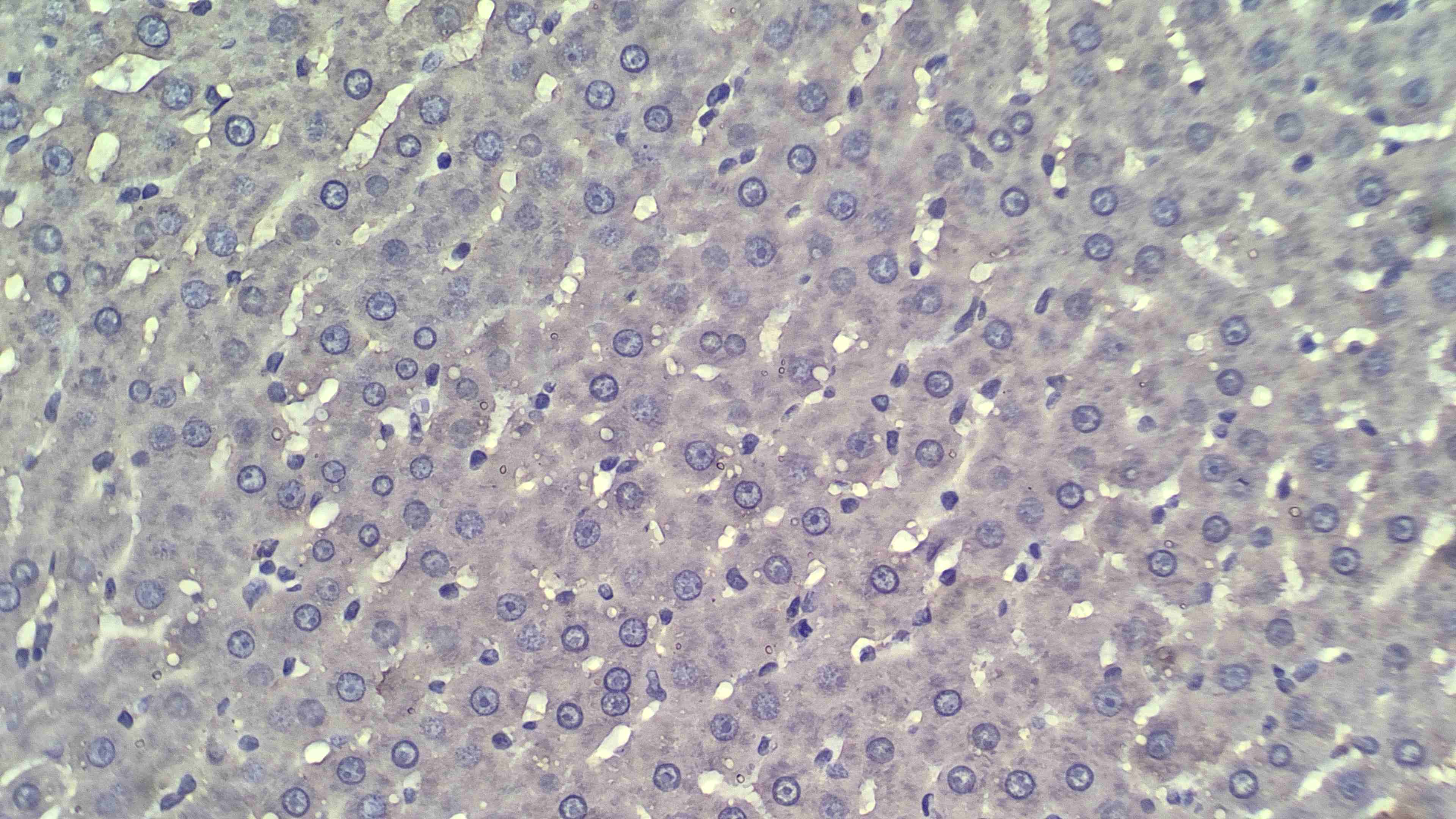

Supplement: Supplementary file 1 [file jox-16-00121-s001.zip › Figure S3 The original images of figure 6/Control.JPG]

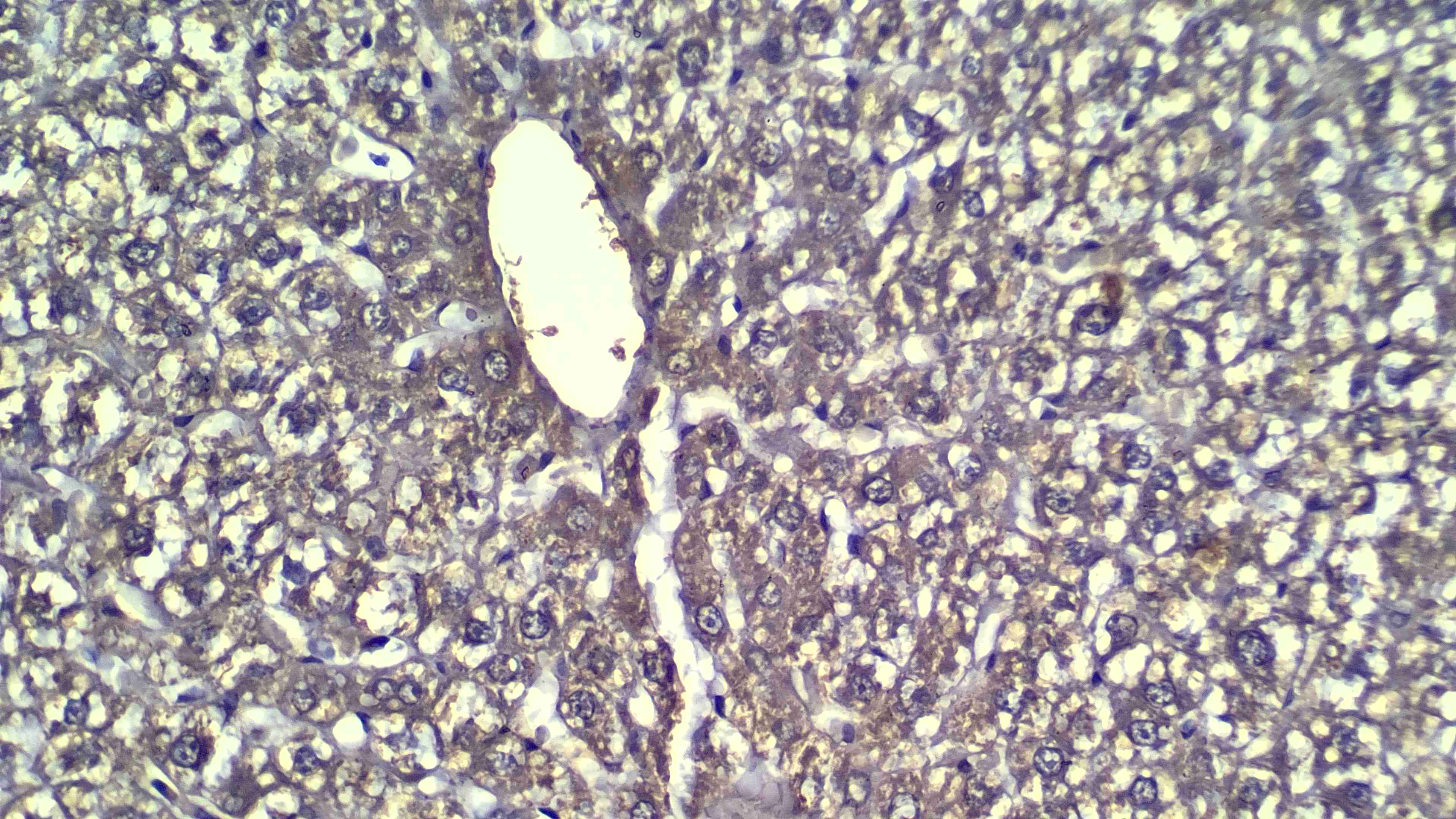

Supplement: Supplementary file 1 [file jox-16-00121-s001.zip › Figure S3 The original images of figure 6/DEXA.JPG]

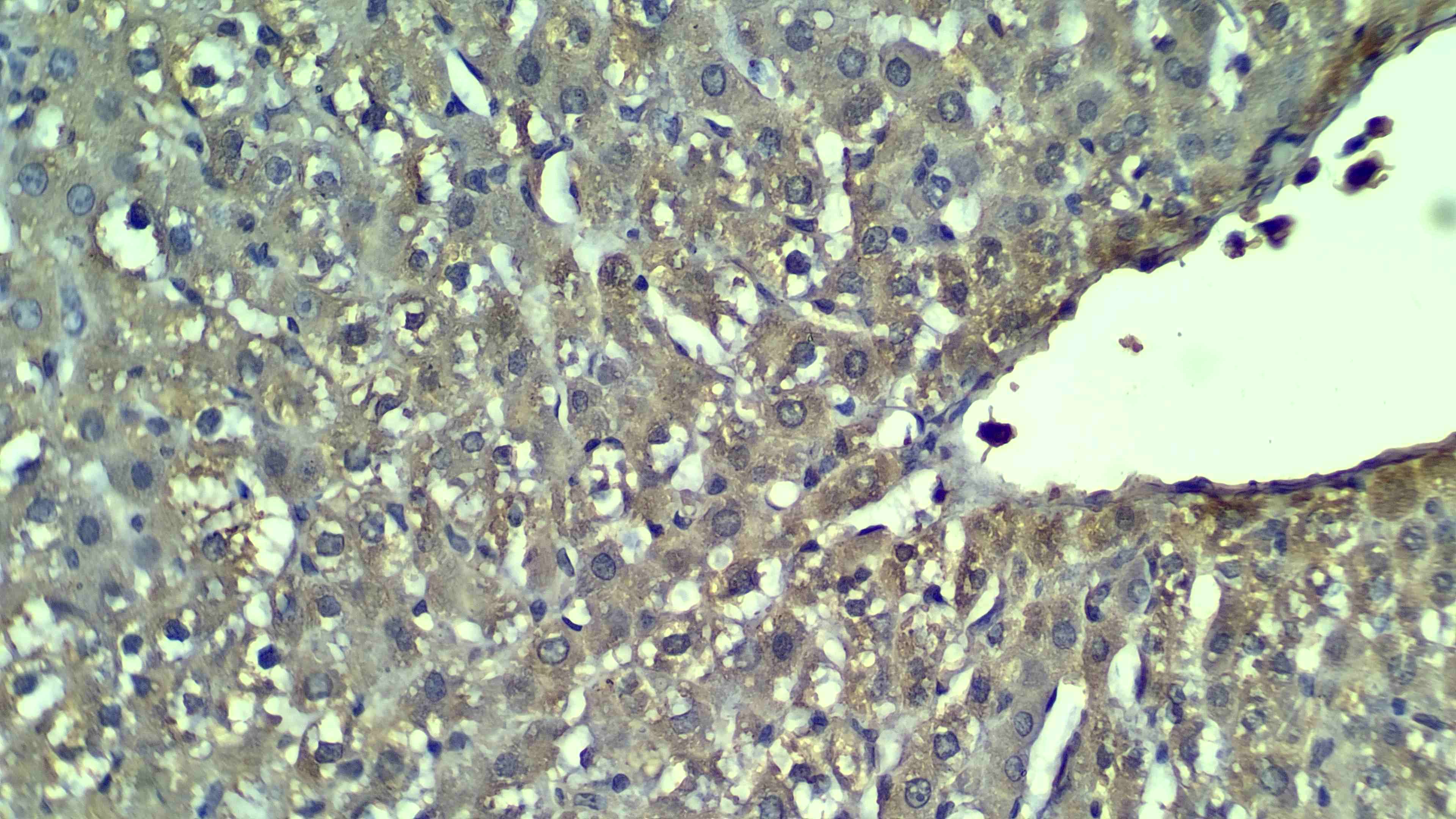

Supplement: Supplementary file 1 [file jox-16-00121-s001.zip › Figure S3 The original images of figure 6/MSM200_DEXA.JPG]

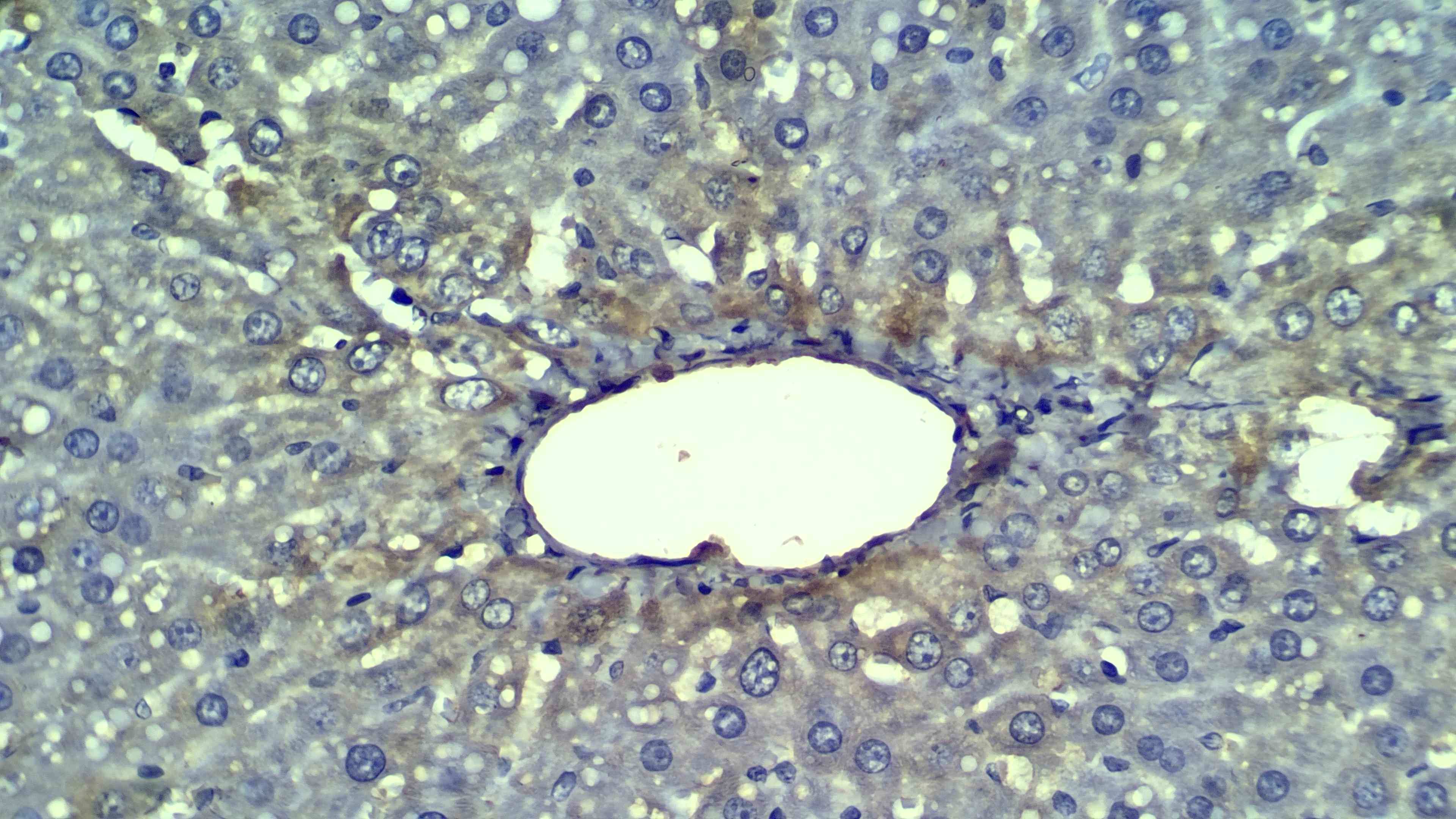

Supplement: Supplementary file 1 [file jox-16-00121-s001.zip › Figure S3 The original images of figure 6/MSM400+DEXA.JPG]

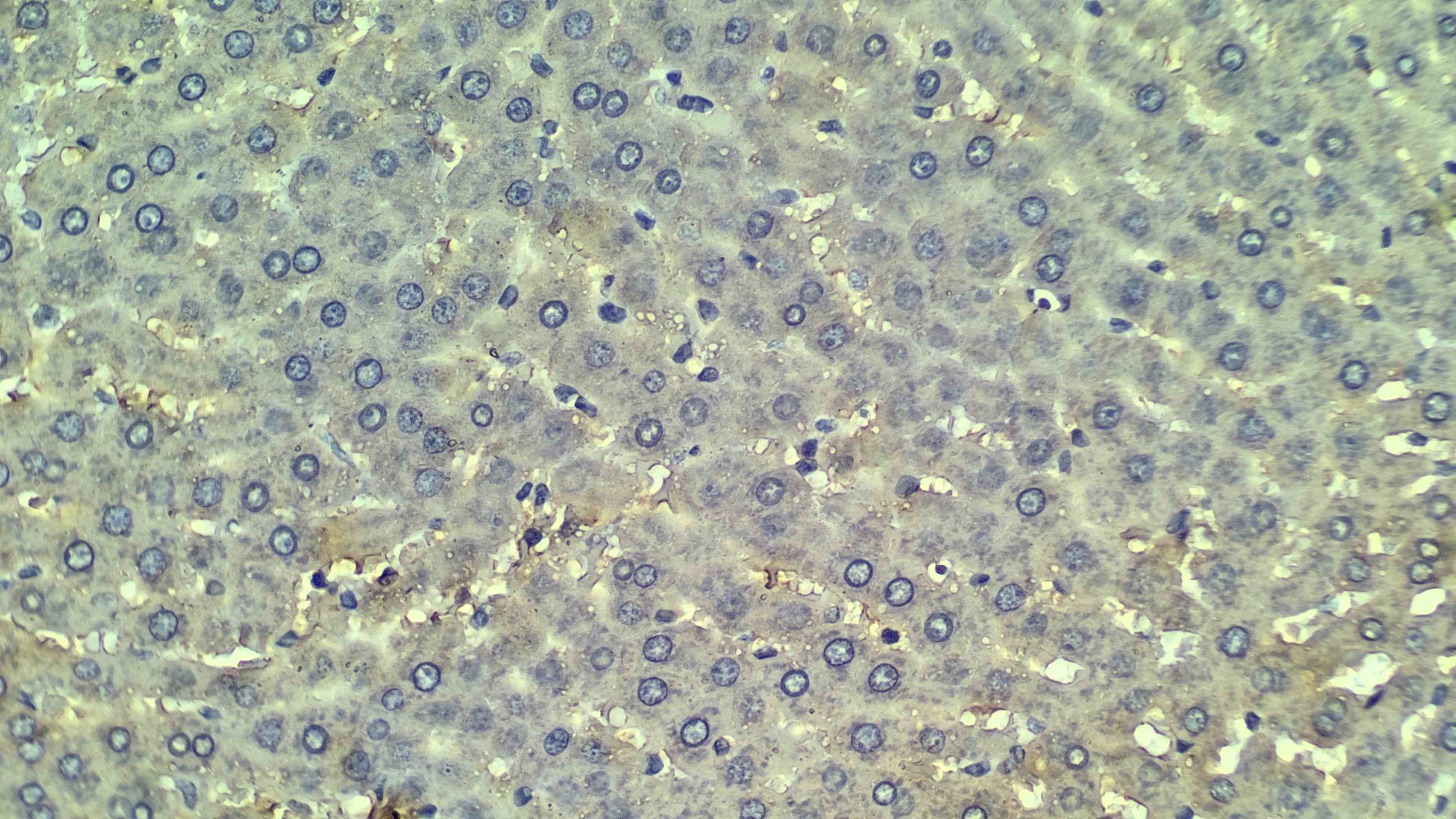

Supplement: Supplementary file 1 [file jox-16-00121-s001.zip › Figure S3 The original images of figure 6/MSM400.JPG]

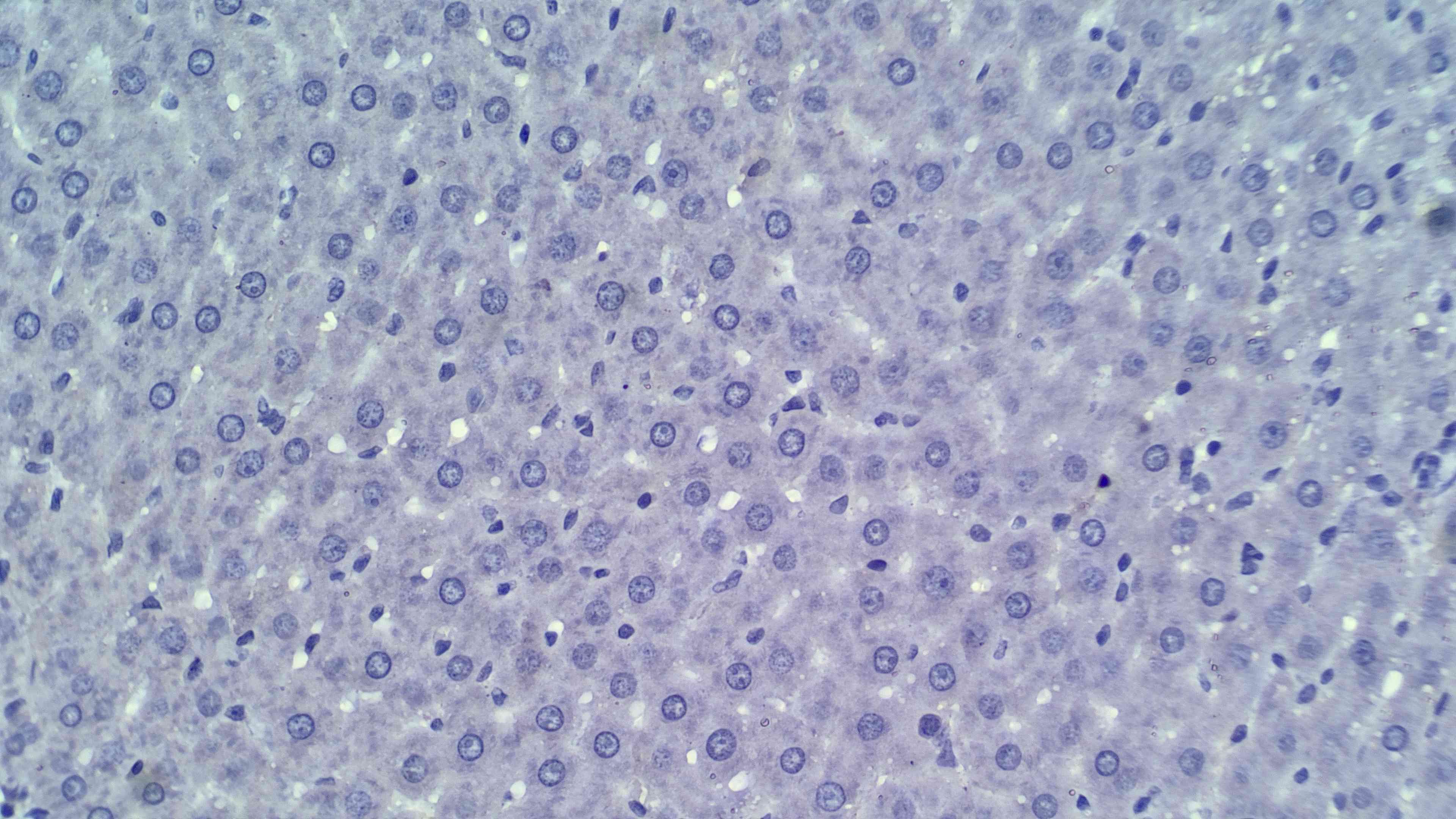

Supplement: Supplementary file 1 [file jox-16-00121-s001.zip › Figure S4 The original images of figure 8/Control.JPG]

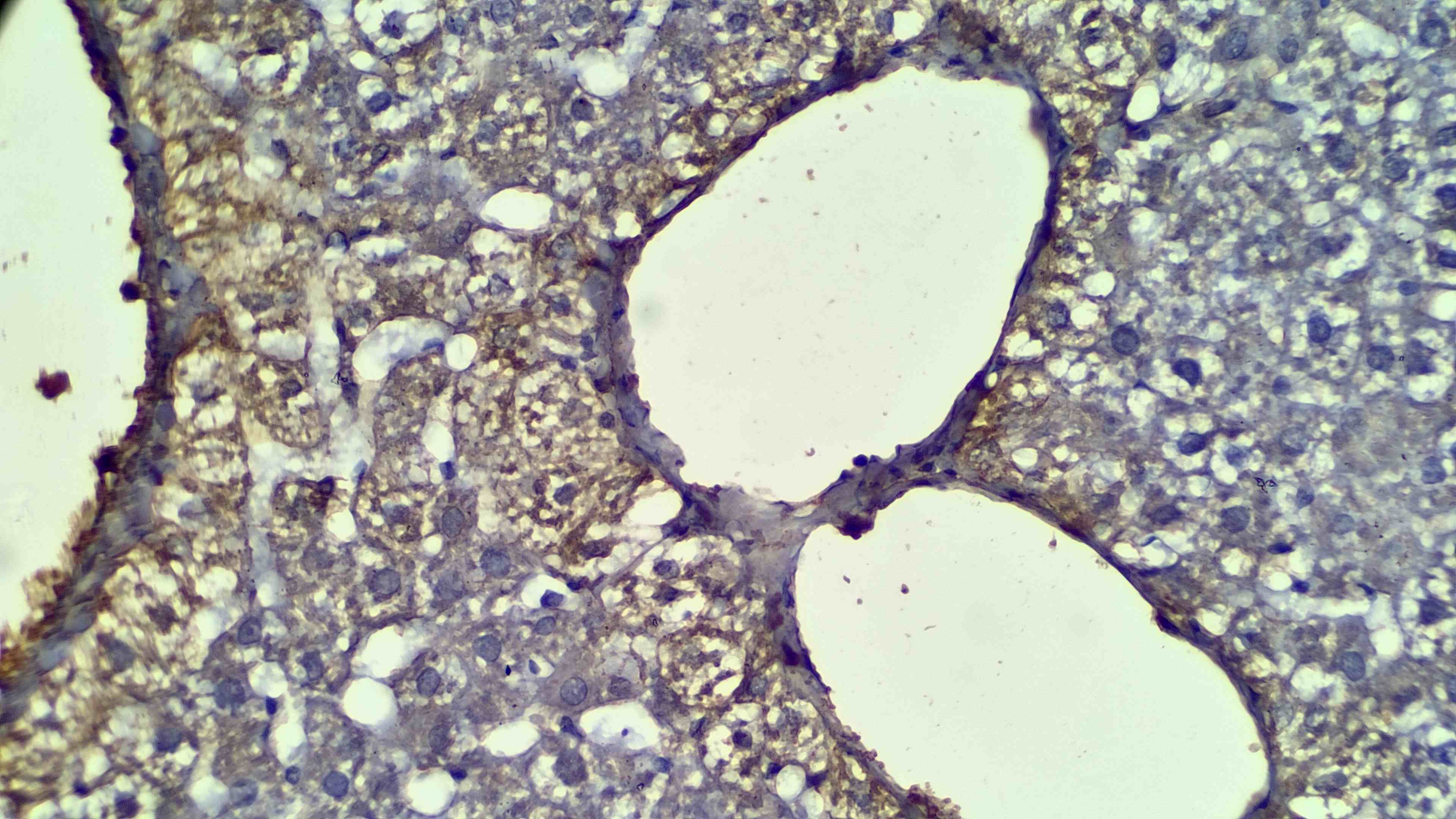

Supplement: Supplementary file 1 [file jox-16-00121-s001.zip › Figure S4 The original images of figure 8/DEXA.JPG]

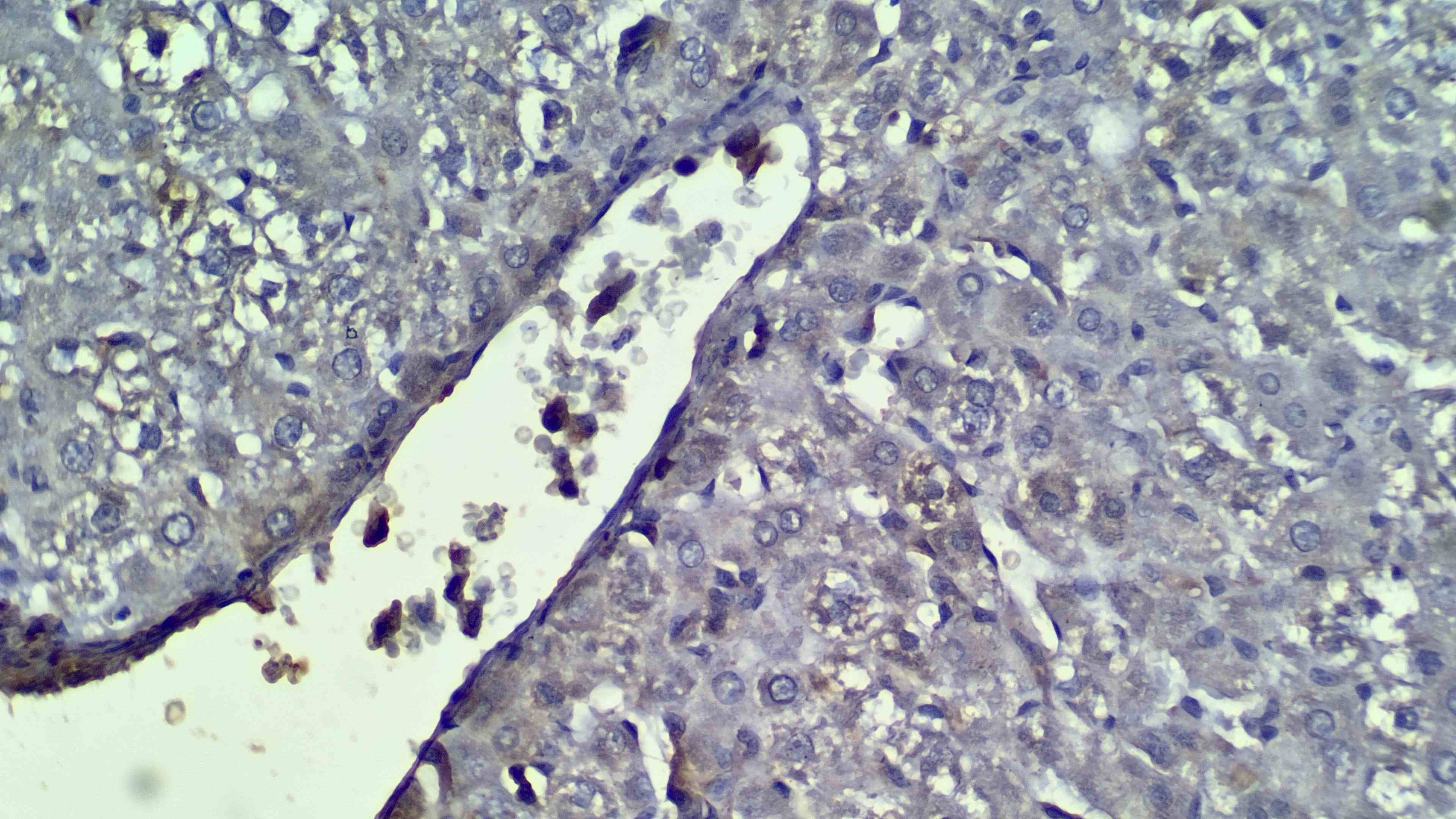

Supplement: Supplementary file 1 [file jox-16-00121-s001.zip › Figure S4 The original images of figure 8/MSM200+DEXA.JPG]

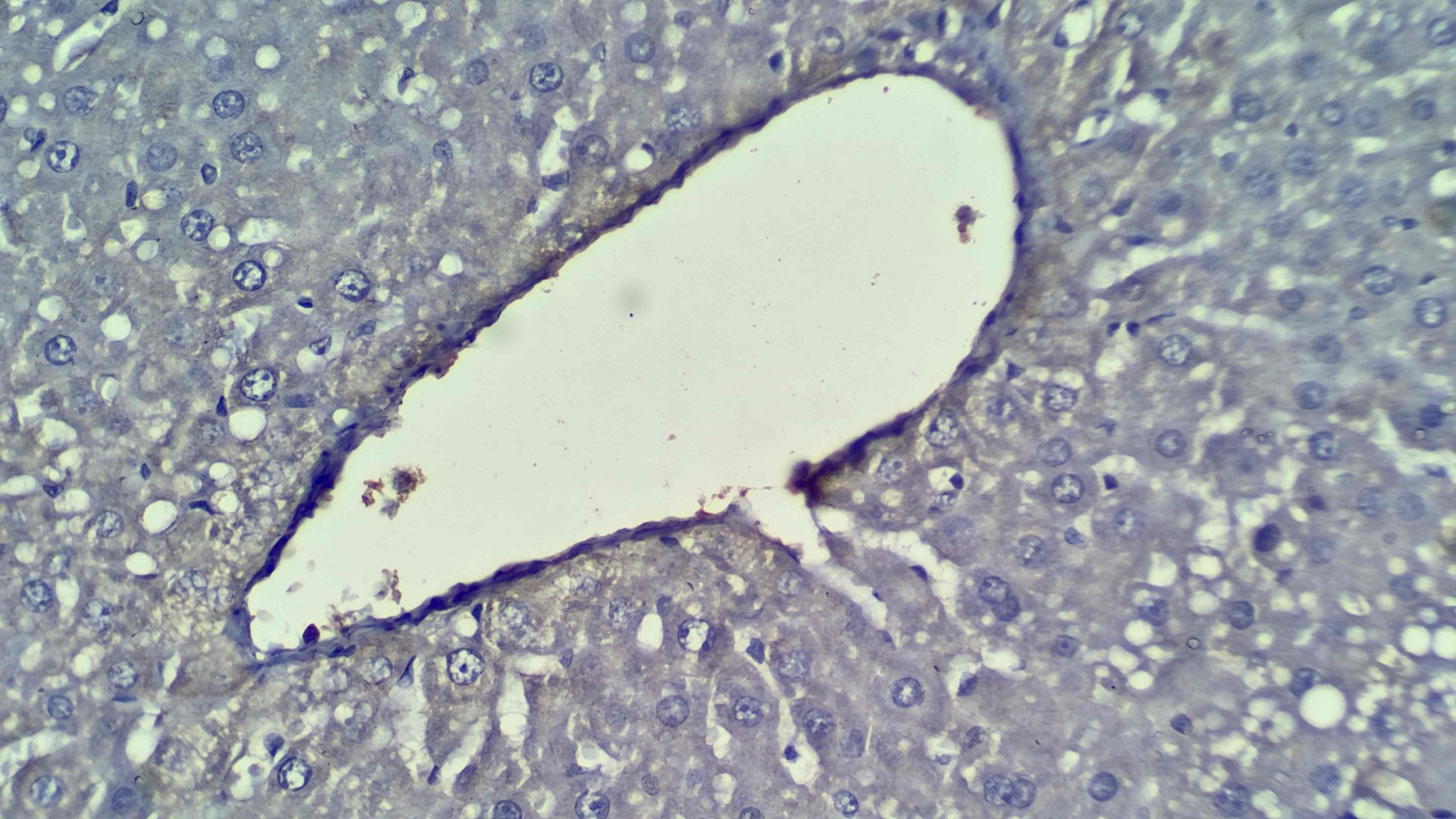

Supplement: Supplementary file 1 [file jox-16-00121-s001.zip › Figure S4 The original images of figure 8/MSM400+DEXA.JPG]

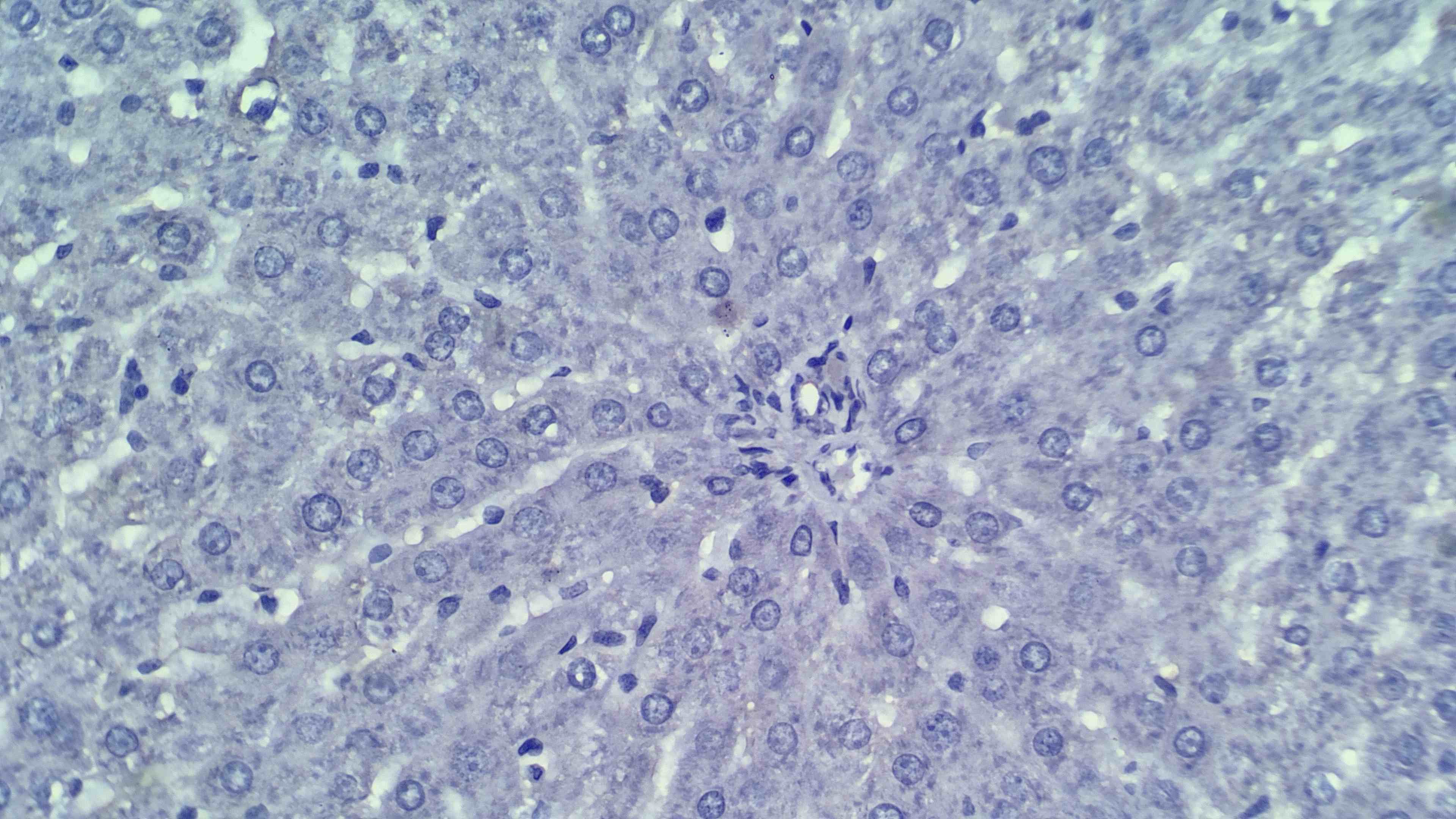

Supplement: Supplementary file 1 [file jox-16-00121-s001.zip › Figure S4 The original images of figure 8/MSM400.JPG]

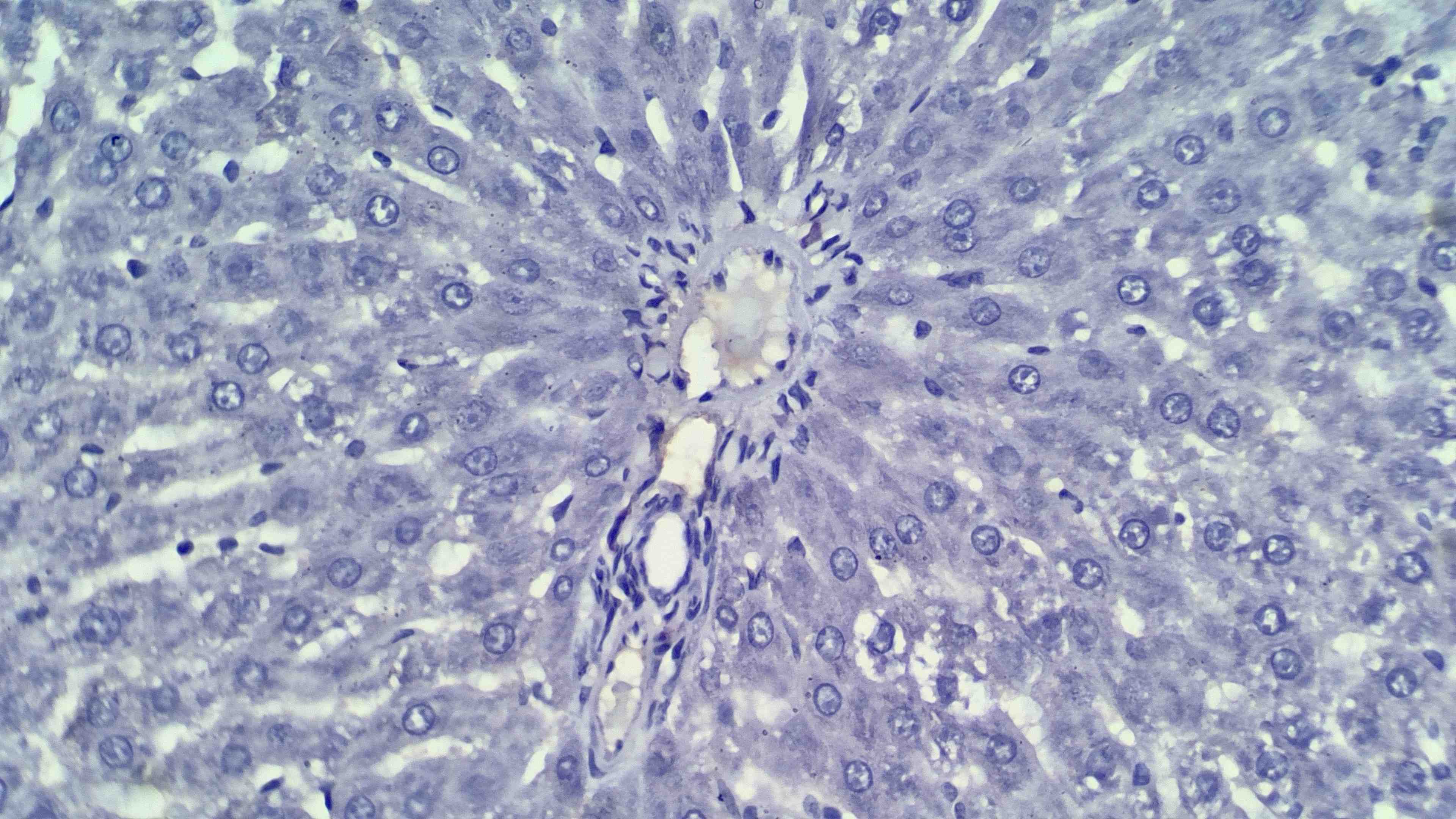

Supplement: Supplementary file 1 [file jox-16-00121-s001.zip › Figure S5 The original images of figure 10/Control.JPG]

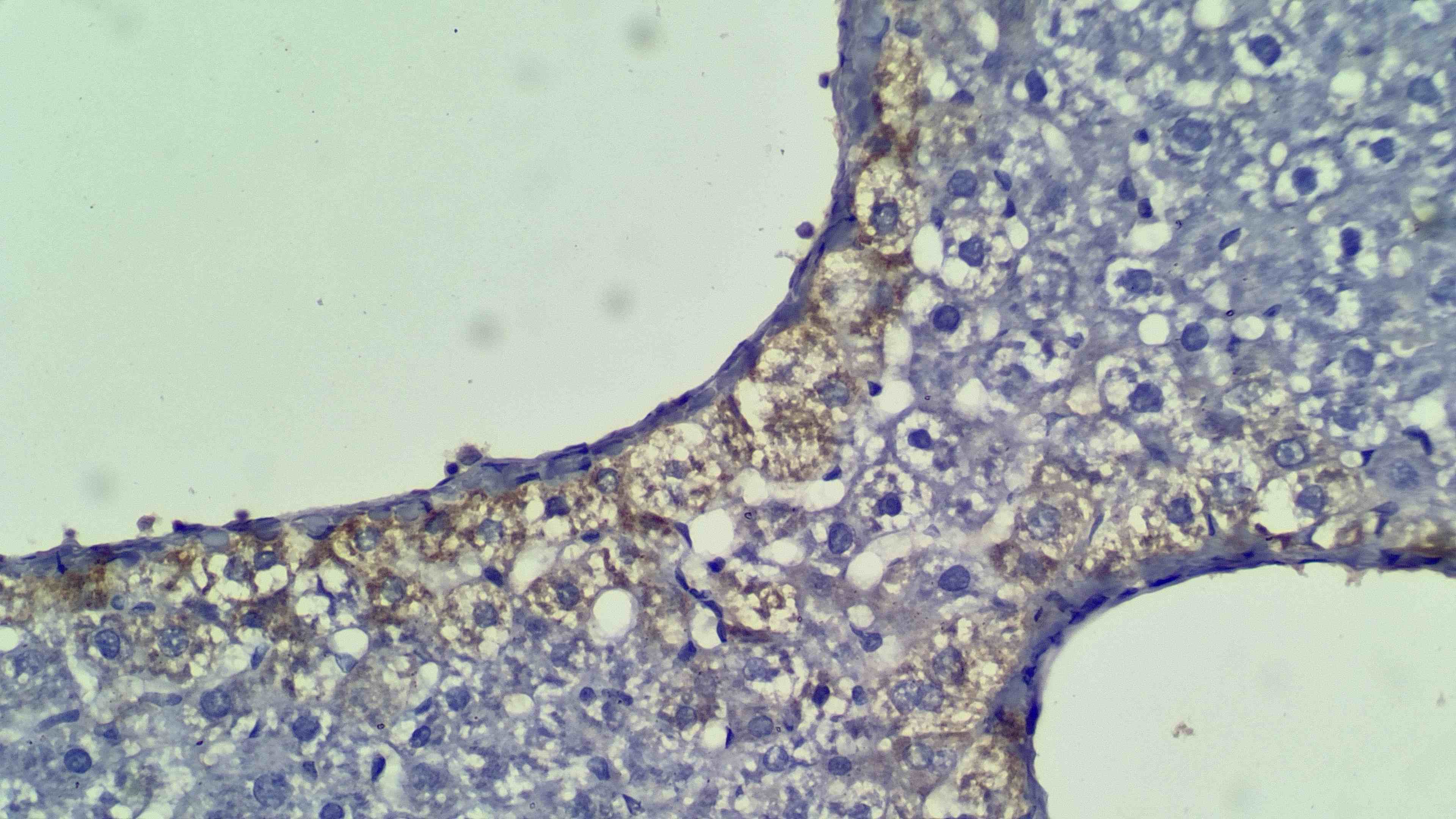

Supplement: Supplementary file 1 [file jox-16-00121-s001.zip › Figure S5 The original images of figure 10/DEXA.JPG]

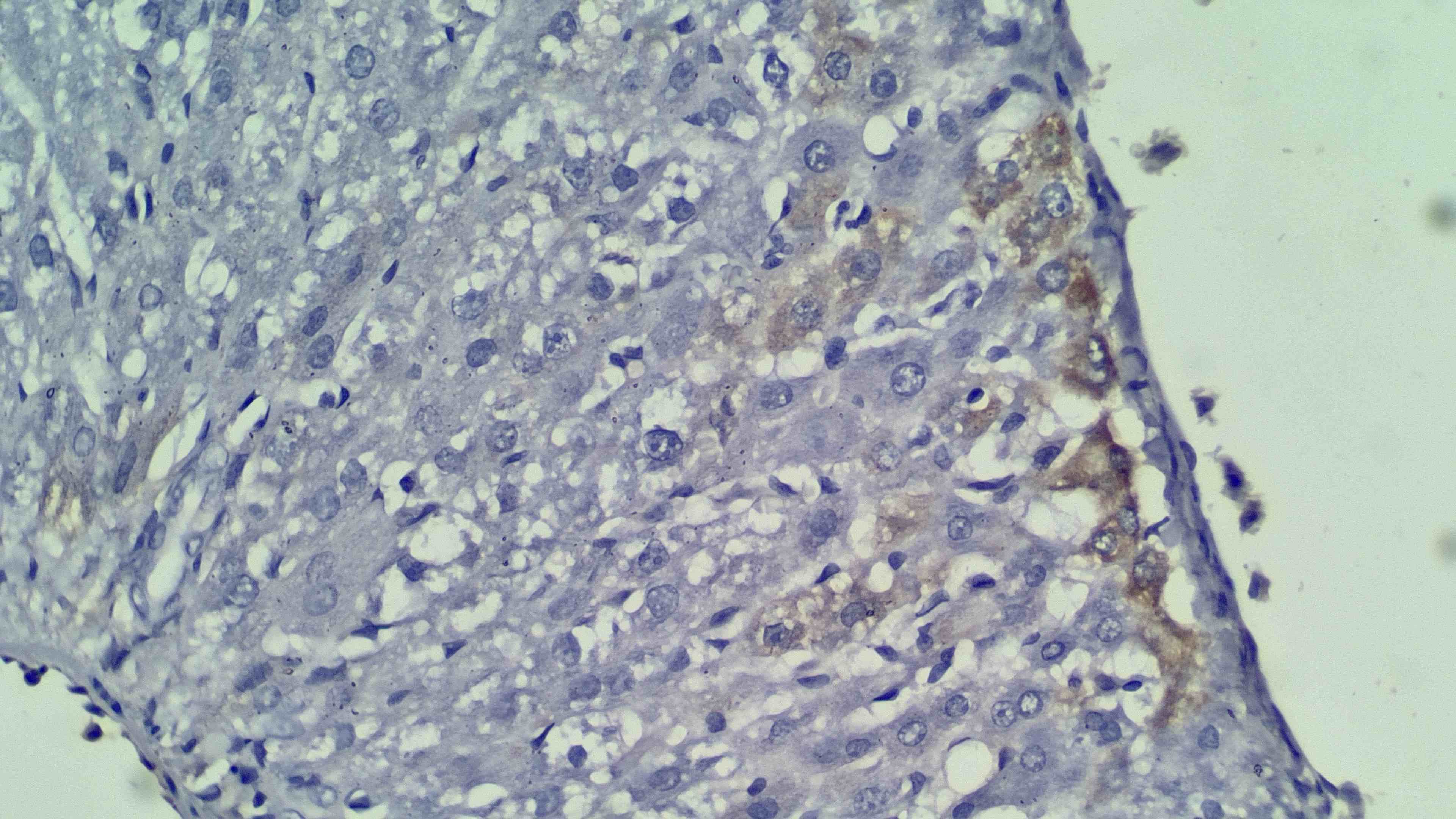

Supplement: Supplementary file 1 [file jox-16-00121-s001.zip › Figure S5 The original images of figure 10/MSM200+DEXA.JPG]

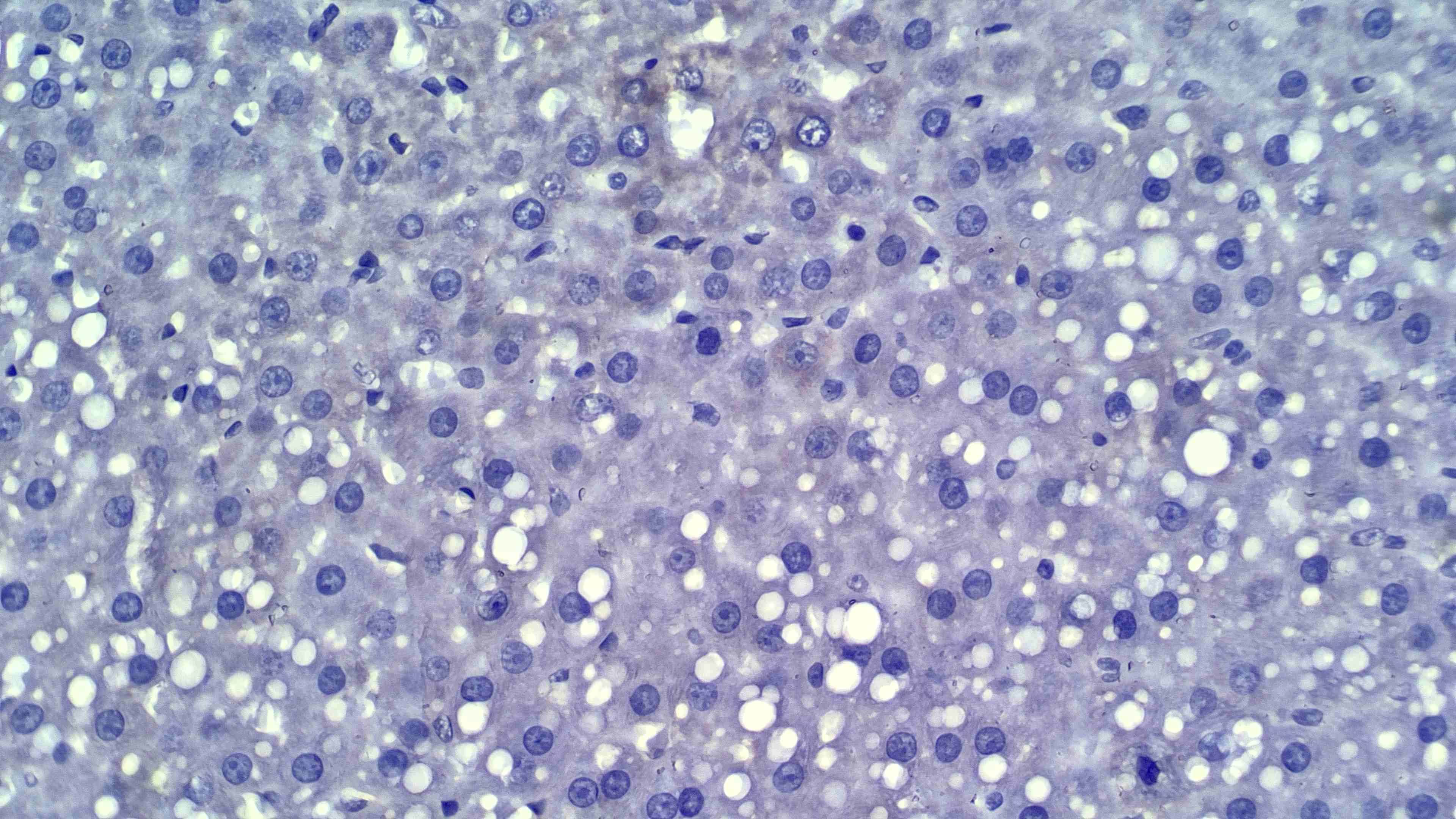

Supplement: Supplementary file 1 [file jox-16-00121-s001.zip › Figure S5 The original images of figure 10/MSM400+DEXA.JPG]

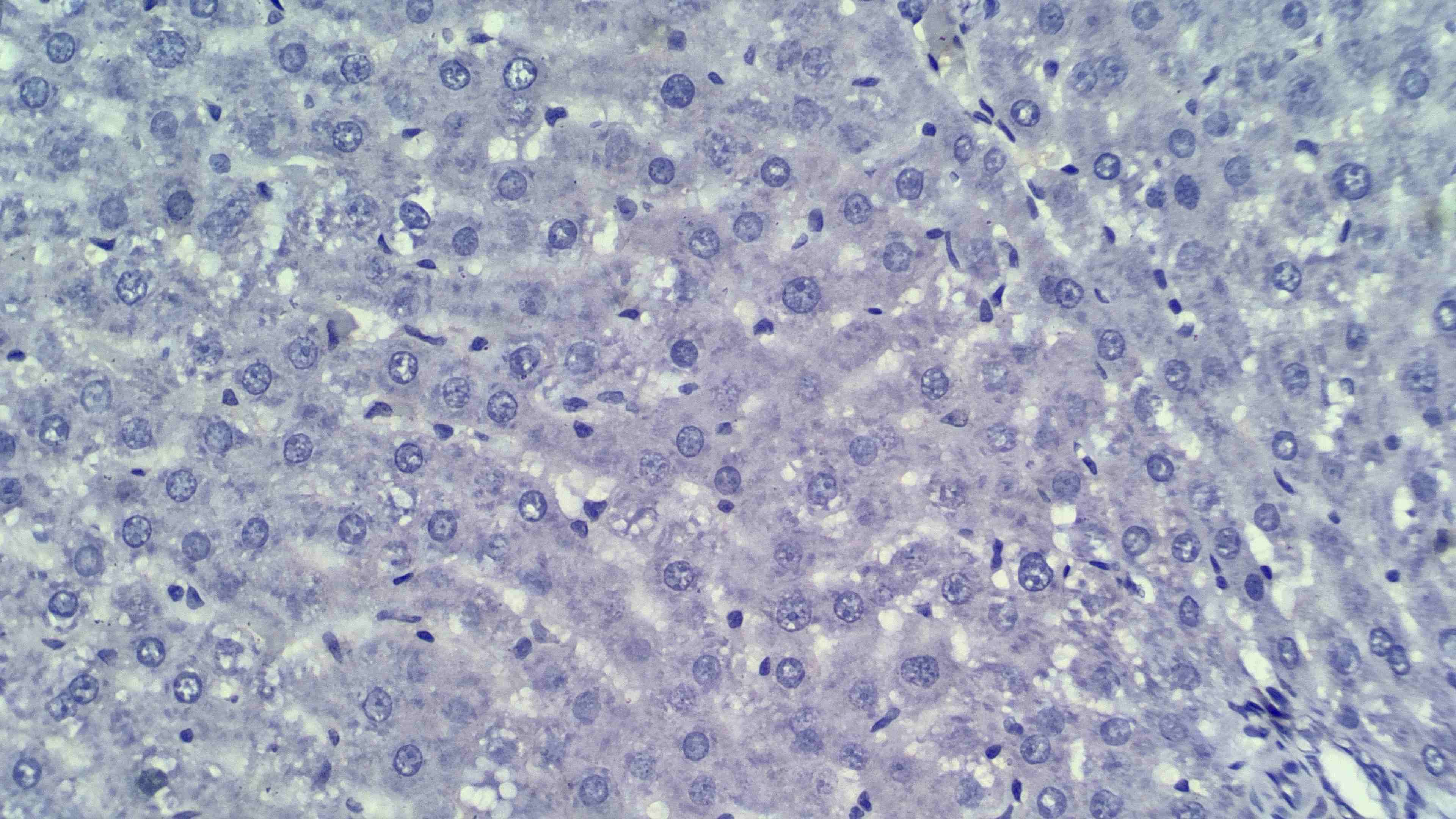

Supplement: Supplementary file 1 [file jox-16-00121-s001.zip › Figure S5 The original images of figure 10/MSM400.JPG]
